# White‐Tailed Deer Baiting Altered Black Bear Site Use but Not Movements or Range Size

**DOI:** 10.1002/ece3.73015

**Published:** 2026-02-12

**Authors:** Nathaniel H. Wehr, Nicholas L. Fowler, Todd M. Kautz, Tyler R. Petroelje, Dean E. Beyer, Jerrold L. Belant

**Affiliations:** ^1^ Pennsylvania Cooperative Fish and Wildlife Research Unit The Pennsylvania State University University Park Pennsylvania USA; ^2^ Alaska Department of Fish and Game Soldotna Alaska USA; ^3^ State University of New York Research Foundation Albany New York USA; ^4^ Michigan Department of Natural Resources Marquette Michigan USA; ^5^ Department of Fisheries and Wildlife Michigan State University East Lansing Michigan USA

**Keywords:** before–after control‐impact, diel activity, GPS collar, home range, recursion, *Ursus americanus*

## Abstract

Intentional anthropogenic food subsidies (i.e., baiting and supplemental feeding) can have profound individual‐level effects on wildlife. We assessed the influence of bait sites targeting white‐tailed deer ( *Odocoileus virginianus* ) on nontarget American black bears ( *Ursus americanus* ) in Michigan's Upper Peninsula, USA. We used black bear GPS collar data collected during July–August 2013–2019 to assess differences in recursive site use metrics (i.e., number, duration, and time between visits) and movement metrics (i.e., range size, step length, and turning angle) using a before–after control‐impact design. We compared site use at 116 baited and 81 unbaited control sites before (pretreatment) and after (treatment) bait applications. We compared movements between 32 GPS‐monitored bear‐years (i.e., individual bears monitored during July–August of each calendar year) where bait was within the bear's home range and eight bear‐years where bait applications occurred outside individuals' home ranges. Bears exhibited 3 times more visits and 11 times longer durations of use at active bait sites during the treatment period than unbaited and pretreatment controls. Active bait site visits were also more nocturnal during the treatment period than visits to unbaited and pretreatment control sites. Movement metrics did not, however, differ between bears with and without bait in their home ranges regardless of treatment period. Our results support the known adaptability of black bears to anthropogenic food subsidies but indicate use of this resource did not influence larger‐scale movement patterns. Our work furthers understanding of black bear behavior by explicitly identifying greater use of baited versus unbaited sites without alteration of movement metrics.

## Graphical abstract

Black bears (*Ursus americanus*) in the Upper Peninsula of Michigan, USA visited sites more often and for longer durations following the application of bait targeting white‐tailed deer (*Odocoileus virginianus*). However, black bear home ranges and movement rates were not influenced by the application of bait.

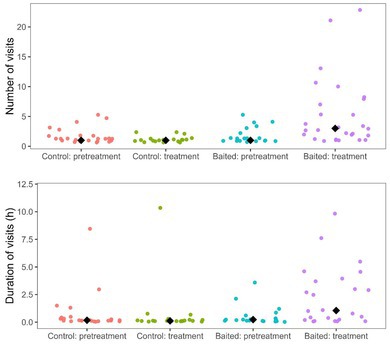

## 1 Introduction

Resource pulses are environmental events where large amounts of a resource become briefly available (Yang et al. 2008). Naturally occurring resource pulses include tree mast events, storm‐driven nutrient run‐offs, and synchronous ungulate births (Yang et al. 2008; Petroelje et al. 2014). Anthropogenic resource pulses include direct (e.g., baiting) and indirect (e.g., hunter harvest remains) food subsidies (Ruth et al. 2003; Candler et al. 2019). Baiting and supplemental feeding are characterized by the intentional attraction of wildlife using food and are commonly used for improving wildlife survival and reproduction, diversion from agricultural or other resources, and recreational viewing or hunter harvest (Putman and Staines 2004; Inslerman et al. 2006; Oro et al. 2013). Wildlife researchers and managers use baiting and supplemental feeding for wildlife capture, monitoring, and delivery of vaccines, contraceptives, and poisons (Linhart et al. 1997; Dunkley and Cattet 2003; Inslerman et al. 2006).

The efficacy of baiting and supplemental feeding varies across species and purposes (Putman and Staines 2004; Oro et al. 2013; Milner et al. 2014). Targeted supplemental feeding can improve wildlife survival (e.g., New England cottontails [ *Sylvilagus transitionalis* ], Weidman and Litvaitis 2011), and targeted baiting can improve wildlife viewing (e.g., brown bears [ *Ursus arctos* ], Penteriani et al. 2017; Milner et al. 2014). Comparatively, the efficacy of targeted baiting to divert wildlife, including American black bears ( *Ursus americanus* ), from other resources or improve hunter harvest is unclear (Conover 2002; Milner et al. 2014; Garshelis et al. 2017). Hunting with and without bait, for example, can produce similar ungulate harvest success rates (Rudolph et al. 2006; Milner et al. 2014). Baiting and supplemental feeding can also negatively influence target (e.g., elk [ *Cervus canadensis* ], Jones et al. 2014) and nontarget (e.g., brown bears, Selva et al. 2017) species through behavioral alterations. Collectively, baiting and supplemental feeding typically influence wildlife positively when implemented by researchers and managers but negatively when implemented by private persons in terms of target and nontarget species welfare (Dubois and Fraser 2013).

The presence of anthropogenic subsidies (i.e., bait and supplemental feed) impacts animal behavior. White‐tailed deer ( *Odocoileus virginianus* ), for example, shifted their core ranges to use bait sites (Williams and DeNicola 2000; Kilpatrick and Stober 2002; Johnson et al. 2021). Deer also fed longer at bait sites than at naturally occurring food sources (Thompson et al. 2008; McCoy et al. 2011). Feral pigs ( *Sus scrofa* ) likewise developed patterns of visiting bait sites nightly after less than 1 week of exposure (Lavelle et al. 2018), and brown bears increased their use of bait sites annually when bait was repeatedly available (Penteriani et al. 2021).

Baiting and supplemental feeding may also influence nontarget species. Remote cameras monitoring white‐tailed deer bait recorded the presence of numerous herbivore, omnivore, and mesocarnivore species (Lambert and Demarais 2001; Bowman et al. 2015). Mammals of all guilds were observed at bait sites using carrion, grains, and other anthropogenic foods to attract brown (Fležar et al. 2019) and black bears (Candler et al. 2019). Nontarget species presence at these anthropogenic feeding sites can be innocuous but can also result in cascading negative effects. For example, the presence of nontarget species at food and water supplementation sites targeting small game species likely did not have consequential effects (Armenteros et al. 2020). Comparatively, rodent use of supplemental feeding targeting red‐backed voles ( *Clethrionomys gapperi* ) was deterred by black bear use (Morris 2005), and predation of artificial hazel grouse ( *Tetrastes bonasia* ) nests was greater near ungulate bait sites because omnivores used both food sources (Selva et al. 2014). These nontarget behavioral modifications may also lead to increased human–wildlife conflicts (Conover 2002; Ruth et al. 2003).

Though baiting and supplemental feeding are important tools for researchers, land managers, and recreationists, consideration of anthropogenic attractants' potential influences on nontarget species is important when developing protocols and policy. We quantified spatial‐behavioral responses of black bears to bait targeting white‐tailed deer. We predicted bear home range sizes and movement rates would decrease in response to baiting as bears concentrated foraging efforts on the readily available and concentrated food source. We also predicted the frequency and duration of bear bait site use would increase when bait was available in comparison to unbaited and untreated sites, again a result of concentrated foraging efforts.

## 2 Materials and Methods

### 2.1 Study Area

**FIGURE 1:**
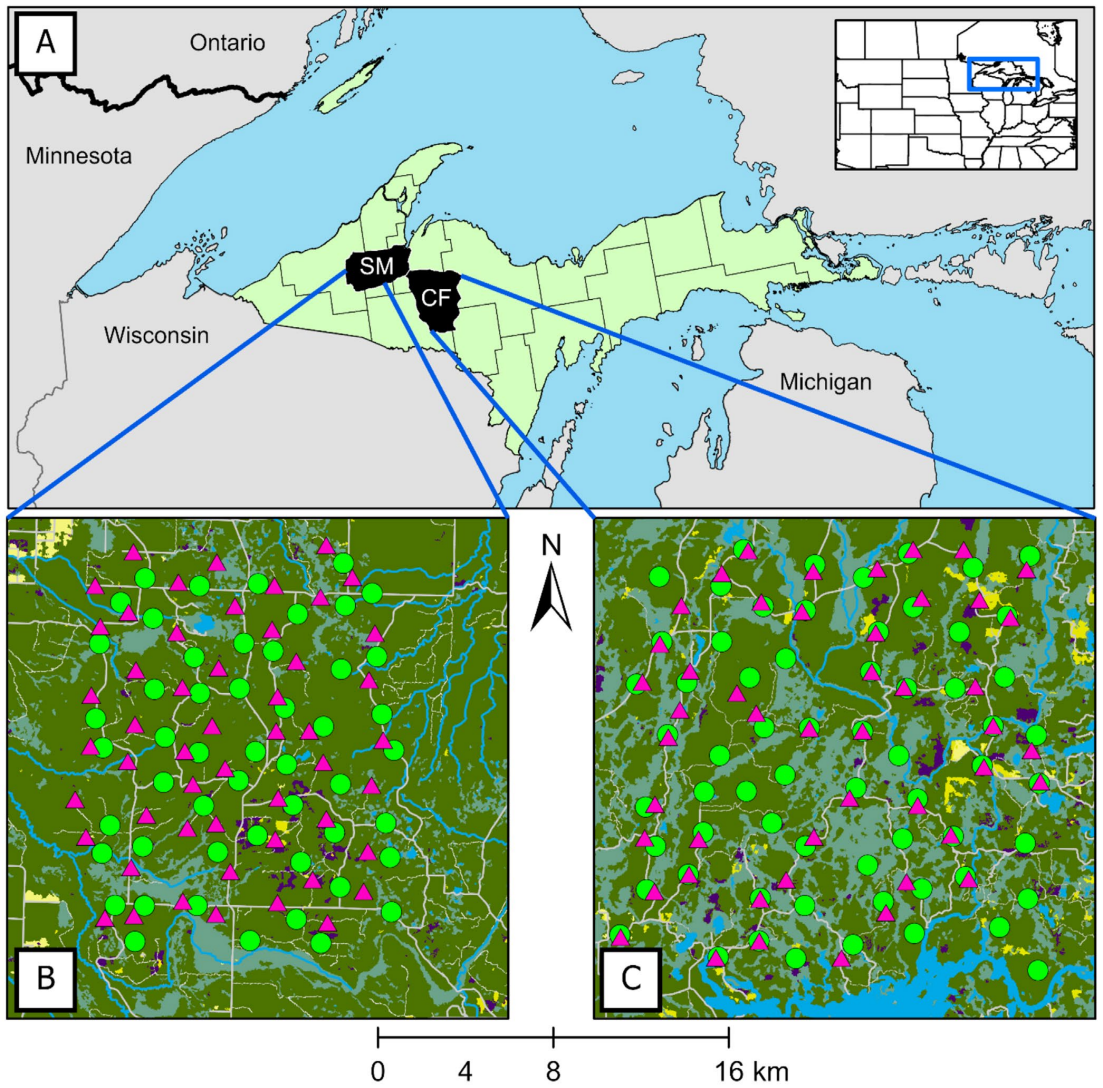
Silver Mountain (SM; 2017–2019) and Crystal Falls (CF; 2013–2015) study areas, Upper Peninsula of Michigan, USA (panel A). Green circles (panels B and C) represent sites baited with whole‐kernel corn, and pink triangles represent unbaited control sites.

We used data from previous studies of predator–prey dynamics in two areas of the Upper Peninsula (UP) of Michigan, USA (e.g., Kautz et al. 2019, 2021; Fowler et al. 2022). During 2013–2015, research was conducted in the Crystal Falls study area (CF; 46.08°–46.59° N, 88.52°–87.92° W), which spanned 1831 km^2^ in Baraga, Dickinson, Iron, and Marquette counties (Figure 1). The CF area was composed primarily of mixed hardwoods (51.5%), including sugar maple ( *Acer saccharum* ), trembling aspen ( *Populus tremuloides* ), black spruce ( *Picea mariana* ), and red pine ( *Pinus resinosa* ) (CCRS et al. 2020; Fowler et al. 2022). Wetlands composed 37.4% of the landscape, with other land covers comprising < 5% each (CCRS et al. 2020). Land ownership was primarily private or commercial forests (82%) (Fowler et al. 2022). Average minimum July temperatures were 10.2°C and maximums were 24.9°C; average August temperature minimums were 9.0°C and maximums were 23.9°C. Average precipitation was 106 and 79 mm in July and August, respectively (NOAA 2015).

During 2017–2019, research was conducted in the Silver Mountain study area (SM; 46.47°–46.79° N, 89.18°–88.43° W). The SM area comprised 1543 km^2^ across Baraga, Houghton, and Ontonagon counties (Figure 1). Forests were composed of sugar maple, trembling aspen, black spruce, white pine ( *Pinus strobus* ), eastern hemlock ( *Tsuga canadensis* ), and northern white cedar ( *Thuja occidentalis* ) covering 79.6% of the study area (CCRS et al. 2020; Fowler et al. 2022). Wetlands covered 9.3% of SM, with other land cover types accounting for < 5% each of the landscape (CCRS et al. 2020). Precipitation averaged 89 mm of rainfall, and average minimum and maximum temperatures were 11.9°C and 23.6°C, respectively, in July (NOAA 2015). In August, average precipitation was 76 mm, average minimum temperature was 11.1°C, and average maximum temperature was 22.7°C (NOAA 2015).

We estimated black bear densities of 39.4/100 km^2^ in CF and 24.2/100 km^2^ in SM using genetic analyses (J. L. Belant, unpublished data). White‐tailed deer were common in the region (adult females: 237 ± 54/100 km^2^; fawns: 334 ± 75/100 km^2^; Kautz et al. 2019). Hunters could legally harvest deer and bear during our study. Bait use was legal for hunting both species. Baiting deer typically began in mid‐September, and bear baiting began in early August (MDNR 2015, 2019). Bait and lure laws did not vary substantially during our study. In the UP, 70% of deer hunters used bait, and 79% approved of hunting with bait (Frawley 2017).

### 2.2 Capture and Monitoring

During May–July of each year from 2013 to 2015 and 2017 to 2019, 101 black bears were captured using barrel traps (*N* = 89) baited with confectionary products, fish, and a scent lure (Stillfried et al. 2015) or modified Aldrich foot snares (Aldrich Animal Trap Co., Clallam Bay, WA, USA) in areas where the deployment of barrel traps was not possible (*N* = 12). Foot snares were equipped with tension relief springs and swivels to reduce impacts. Captured bears were immobilized with 4–7 mg/kg Telazol injected via pole syringe (*N* = 99) or dart gun (*N* = 2; Stillfried et al. 2015). For each captured bear, researchers first assessed potential injury and trauma associated with capture, but no injuries were reported beyond localized swelling from foot snares. Researchers then recorded morphometric measurements, sex, and evidence of dependent young (i.e., females that were lactating or accompanied by cubs‐of‐the‐year) and fitted the bear with a Lotek model GPS7000SU global positioning system (GPS) collar (Lotek Wireless, Newmarket, ON, Canada). Techniques for animal capture and handling were approved by university institutional animal care and use committees (IACUC; Mississippi State University: IACUC 12–012, 15–013, and 17–119; State University of New York College of Environmental Science and Forestry: IACUC 180501) and a state‐certified veterinarian as described previously (Kautz et al. 2021). Captures were conducted by IACUC‐approved personnel on private property with permission from landowners, state property with permission from MDNR, and federal property with permission from the U.S. Forest Service. Collars attempted a GPS relocation every 15 min. Bears were monitored during July–August during each study year: 2013–2015 and 2017–2019. Researchers uploaded location data from collars via fixed‐wing aircraft 1–2 times per week and did not observe any mortalities of collared individuals during the annual monitoring periods. Collars were removed from bears during den checks at the conclusion of the study or recovered from bears following mortality events.

### 2.3 Bait Sites

Prior studies maintained 64 bait sites in CF during 2013–2015 and 52 bait sites in SM during 2017–2019 (Kautz et al. 2019). In each study area, researchers created a hexagonal grid (6.25 km^2^ cells) and established a bait site in each grid cell. Bait sites were within 200 m of an accessible road or trail and sites in adjacent cells were ≥ 2 km apart (Figure 1). Whenever possible, bait sites were on level ground in deciduous forest near evidence of white‐tailed deer. Each year, researchers applied bait on 11 or 12 August (Day 0), reapplied bait on Days 3, 6, 10, 14, and 18, and monitored each site for activity using remote cameras through Day 21. Each bait application comprised 5.5 kg of whole‐kernel corn distributed in a 2–3‐m‐diameter circle to simulate baiting by deer hunters.

Using the same criteria, researchers selected 29 unbaited control sites in CF and 52 unbaited control sites in SM. Control sites were selected and monitored for activity using remote cameras during 2017–2019. Control sites were located > 200 m from baited sites so that black bears could not simultaneously occupy both locations (median = 919 m, range = 203–1991 m). Unbaited control sites were not included for all baited sites due to limited accessibility.

Pretreatment periods began during 15–23 July (except in 2014 when pretreatment began 4 August). The pretreatment period consisted of the 8–26‐day period before initial bait application. Treatment periods began on 11–12 August each year, coinciding with initial bait applications. The treatment period consisted of the 21‐day period following initial bait application.

### 2.4 Data Analysis

The objective of our data analysis was to assess the spatial‐behavioral response of black bears to the application of baiting using GPS‐monitoring data. We completed all data curation and analyses in R (v4.4.2; R Core Team 2024; Appendix S1). Our sample unit was individual bear‐years (i.e., GPS‐monitoring data from individual bears monitored each year with bears monitored multiple years considered distinct sample units each year). From our initial sample of 53 bear‐years, we excluded 10 bear‐years due to insufficient data (e.g., collars acquired locations intermittently or stopped acquiring locations prematurely). We also excluded three bears that emigrated during the study period and therefore did not have stable home ranges. We identified emigration using variograms of directional movement in the *ctmm* package; variograms assess space use relative to a starting point (Calabrese et al. 2016; Fleming and Calabrese 2023). We considered bears to be actively emigrating if variograms of their movement did not reach a stable plateau. For the 40 remaining bear‐years, we estimated 95% kernel density estimate (KDE) home ranges as calculated for the entire study period (i.e., inclusive of the pretreatment and treatment periods) using the *adehabitatHR* package (Calenge 2006, 2024). From these, we categorized bear‐years as those with (*n* = 32 bear‐years) or without (*n* = 8 bear‐years) home range overlap with bait sites. We then categorized locations as occurring before (pretreatment) or after (treatment) bait application began.

We applied a before–after control‐impact (BACI) design for our analysis (Smith 2002). Corresponding to our first prediction, we assessed black bear movement metrics (i.e., home range size, core range size, median step length, and median turning angle) during the pretreatment and treatment periods and considered bears without bait in their KDE home ranges the control and bears with bait inside their KDE home ranges the impact group. We estimated movement metrics using the *adehabitatHR* package (Calenge 2006, 2024) similarly to Petroelje et al. (2019). For each bear each year, we estimated home and core range areas using 95% and 50% KDE from locations during the pretreatment and treatment periods (Petroelje et al. 2019). We estimated median step length using all consecutive paired locations (i.e., locations recorded without temporal gaps) and median turning angle using the absolute value of the relative turning angle between three consecutive locations in radians (Petroelje et al. 2019). Smaller home and core range sizes as well as more torturous movements correspond to more concentrated resource use.

Corresponding to our second prediction, we assessed bait site activity (i.e., number of visits, duration of visits, interval between visits, and diel time of visits) during the pretreatment and treatment periods. We considered bait site visits to begin when a black bear was within 100 m of a site and conclude when an individual left the area defined by the radius. We used a 100‐m threshold to ensure multiple consecutive recorded locations would occur within the threshold distance (Bracis et al. 2018). We then derived metrics of bait site activity from these values using recursion analysis from the *recurse* package (Bracis et al. 2018). Recursion analyses estimate site entrance and exit times using an interpolated estimate of the proportion of a step occurring at each bait site.

We conducted our BACI analysis using generalized linear mixed models (GLMMs) with the *glmmTMB* package (Brooks et al. 2017). We fit seven response variables (home range, core range, step length, turning angle, visits, duration, and interval) using baiting period (i.e., pretreatment or treatment), individual (i.e., bait within or outside home range) or site (i.e., baited or unbaited control), and the interaction between period and individual or site as explanatory variables following a standard BACI design (Smith 2002). We considered bear ID as a random effect in movement metric models and site ID as a random effect in site activity models. We selected the best‐fitting distribution for each model using a Pearson *Χ* ^2^ test of the residuals using the *DHARMa* package (Hartig 2024). We fit home range, core range, and interval using a gamma distribution with a log‐link function, step length and turning angle using a Gaussian distribution, and visits and duration using a negative binomial distribution with a log‐link function. In lieu of the BACI design used for other predictor variables, we used Watson's two‐sample test of homogeneity to compare distributions of diel activity at control sites and treatment sites, respectively, before and after baiting using the *circular* package (Agostinelli and Lund 2024). We also assessed temporal overlap (i.e., the two‐dimensional overlap of activity distributions: ${\hat{\Delta }}_{1}$) using the *overlap* package (Schmid and Schmidt 2006; Meredith et al. 2024).

## 3 Results

We analyzed movement data from 31 individual black bears (20 females, 11 males) consisting of 140,977 locations recorded during July–August across 40 bear‐years (median = 3776 locations per bear year; range = 1803–4419). Of these locations, 71,665 occurred before baiting (median = 1978; range = 430–2550) and 69,312 occurred after baiting (median = 1852; range = 593–1985). Of analyzed locations, 114,172 locations were recorded for bears with bait inside their KDE home ranges (median = 3776; range = 1803–4419), and 26,805 locations were recorded for bears whose KDE home ranges did not include bait sites (median = 3810; range = 2440–3925). Bears with bait sites in their KDE home ranges included 15 females and 8 males. Bears without bait sites in their KDE home ranges included 5 females and 3 males.

**FIGURE 2:**
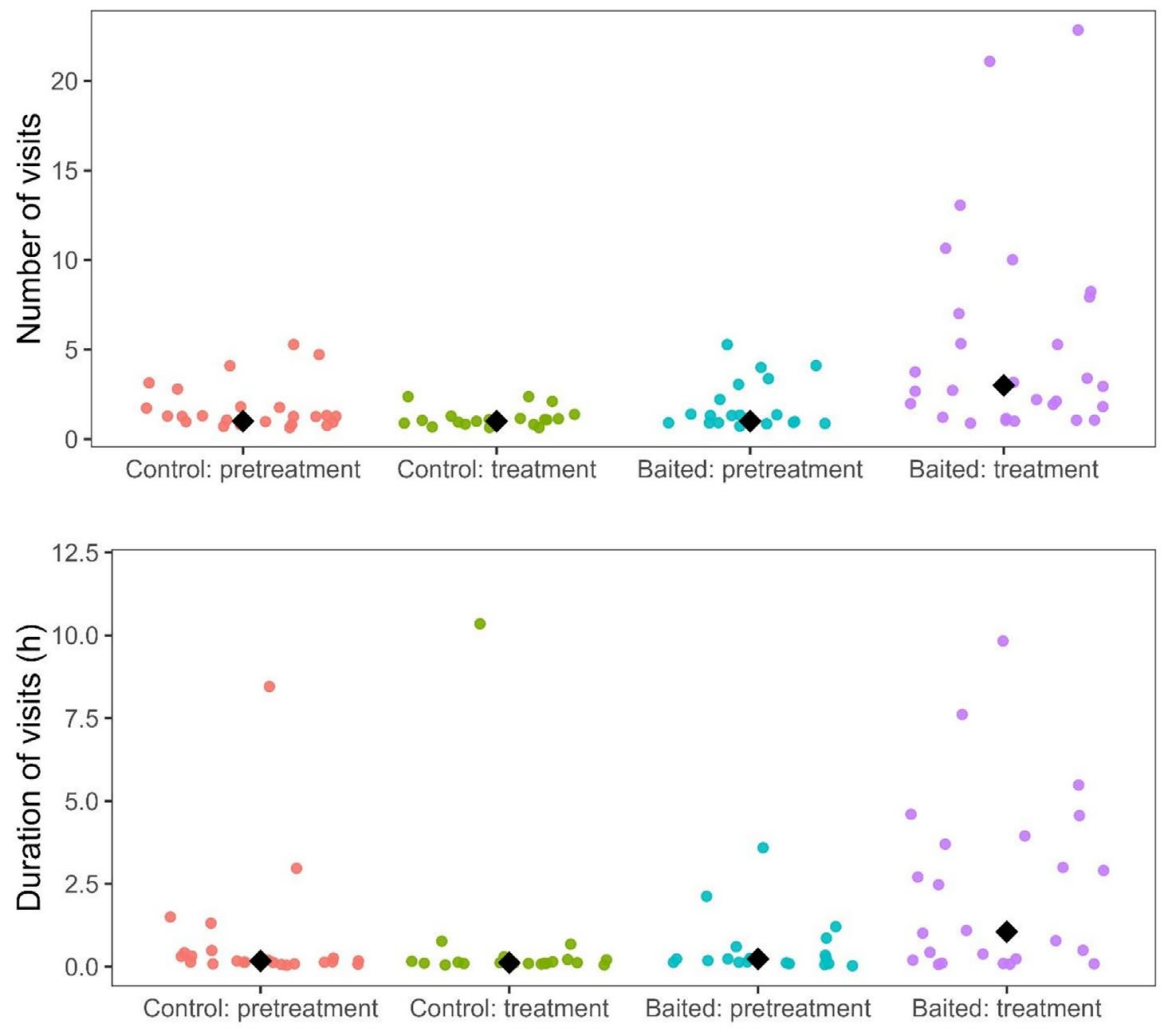
Number and duration of visits by American black bears ( *Ursus americanus* ) at sites before (pretreatment) and after (treatment) baiting (whole‐kernel corn) or unbaited control sites, Upper Peninsula of Michigan, USA, 2013–2019. Colored circles represent values for individual sites using a before–after control‐impact design. Black diamonds represent median values.

**FIGURE 3:**
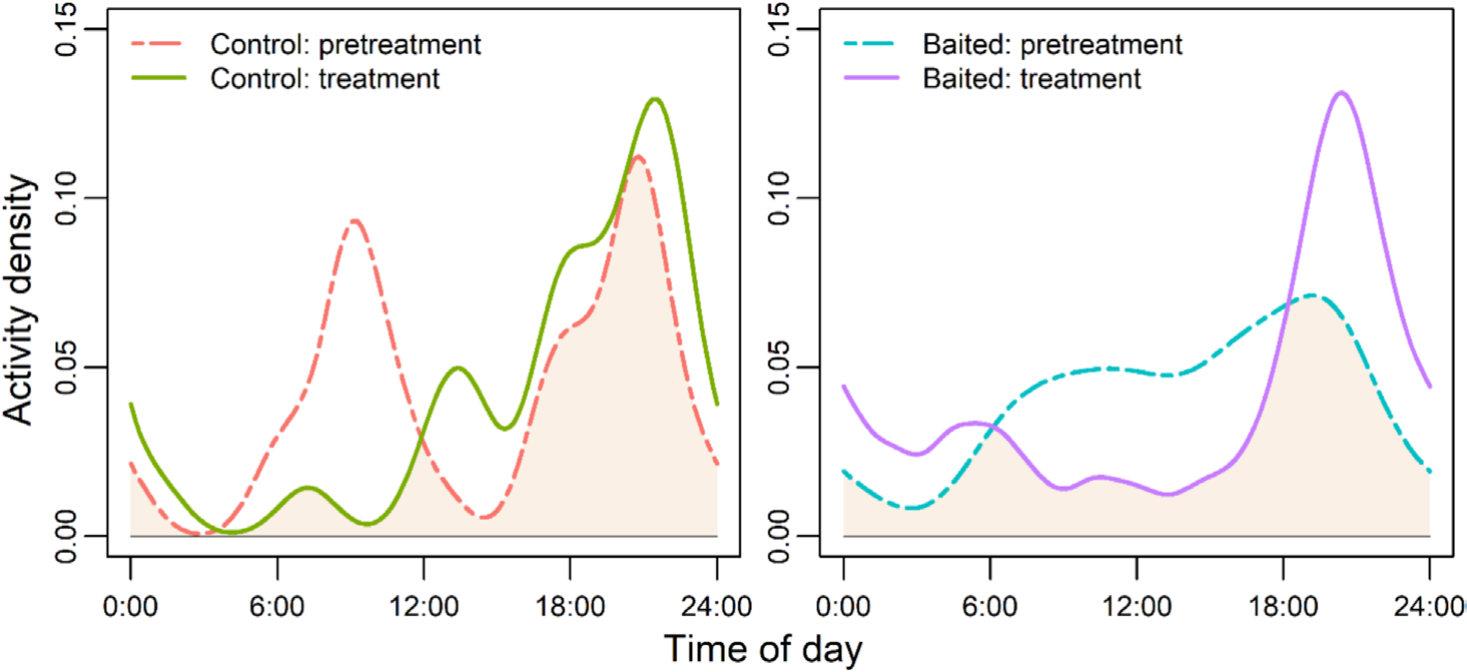
Diel activity by American black bears ( *Ursus americanus* ) at sites before (pretreatment) and after (treatment) baiting (whole‐kernel corn) or unbaited control sites, Upper Peninsula of Michigan, USA, 2013–2019. Colored lines represent the activity density of site visits using a before–after control‐impact design.

Black bear home range size, core range size, and median step lengths were not statistically different among bears during the pretreatment and treatment periods regardless of bait site presence within their home ranges (Table 1). Median turning angles were smaller among bears with bait sites in their home ranges (*β* = −0.15 ± 0.06, *p* = 0.01), but median turning angle was not influenced by treatment period (*β* = −0.06 ± 0.06, *p* = 0.37). Bears visited baited sites 3 times more frequently and spent 11 times greater durations at baited sites than pretreatment and unbaited control sites, but the time between consecutive visits did not differ between treatment periods (Figure 2; Table 1). Bear activity was more nocturnal at bait sites during the treatment period (Figure 3; *U* ^2^ = 0.44, *p* < 0.01) and did not differ at control sites (*U* ^2^ = 0.11, *p* > 0.1), though temporal overlap remained high in both comparisons (${\hat{\Delta }}_{1}$ = 0.67 and ${\hat{\Delta }}_{1}$ = 0.65, respectively).

**TABLE 1:** Estimates and model coefficients for American black bear ( *Ursus americanus* ) movement metrics and recursive use at sites before (pretreatment) and after (treatment) baiting, Upper Peninsula of Michigan, USA, 2013–2019. Black bears with (*n* = 32 bear‐years) and without (*n* = 8 bear‐years) bait (whole‐kernel corn) access were defined using home range overlap with known bait sites used for white‐tailed deer ( *Odocoileus virginianus* ) research. Unbaited control sites (*N* = 81) were located near baited sites (*N* = 116; Figure 1). Estimates represent medians (with standard errors) for respective metrics. *β*, *z*, and *p* are derived from the interaction of period and site from before–after control‐impact (BACI) models.

Metric	Without access to bait (*n* = 8)		With baited site estimates (*n* = 32)		BACI interaction result		
	Pretreatment	Treatment	Pretreatment	Treatment	*β*	*z*	*p*
Home range (km^2^)	30.0 (18.0)	33.8 (106.9)	39.0 (106.0)	91.7 (180.6)	−0.26 ± 0.64	−0.41	0.69
Core range (km^2^)	8.3 (4.0)	6.5 (17.2)	7.2 (23.0)	20.8 (39.0)	−0.33 ± 0.59	−0.55	0.58
Step length (m/15 min)	37.9 (2.4)	29.5 (3.5)	48.3 (6.5)	31.9 (4.3)	10.70 ± 6.13	1.75	0.08
Turning angle (rad)	1.32 (0.07)	1.39 (0.07)	1.13 (0.04)	1.34 (0.05)	−0.06 ± 0.06	−1.06	0.29

Metric	Unbaited site estimates (*N* = 81)		Baited site estimates (*N* = 116)		BACI interaction result		
	Pretreatment	Treatment	Pretreatment	Treatment	*β*	*z*	*p*
Number of visits	1 (0.6)	1 (0.2)	1 (0.6)	3 (2.6)	−1.42 ± 0.49	−2.90	< 0.01
Duration (h)	0.17 (0.79)	0.12 (1.02)	0.23 (0.39)	2.59 (4.49)	−2.51 ± 0.92	−2.71	< 0.01
Return rate (day)	2.13 (1.21)	5.18 (0.34)	3.83 (0.72)	0.59 (0.44)	1.27 ± 1.10	1.16	0.25

## 4 Discussion

Black bears in our study visited sites more often and remained at sites for longer durations following baiting with whole‐kernel corn, supporting our predictions. As opportunistic omnivores, black bears concentrate activity in areas of high‐quality food (Samson and Huot 2001; Bowersock et al. 2021), and calorie‐rich anthropogenic food sources can constitute substantial portions of black bears' diets (Dobey et al. 2005; Benson and Chamberlain 2006). In previous studies, anthropogenic foods placed specifically for black bears (Partridge et al. 2001) or available from other sources (Kirby et al. 2017) predominated black bear diets when available. Our results demonstrate increased bait site use by black bears compared with control sites and are consistent with studies showing frequent black bear visitation of baited sites (Bowman et al. 2015; Candler et al. 2019). Similarly, brown bears in Poland (Selva et al. 2017) visited bait sites three times more frequently than unbaited sites. Baiting also increased detections of wild boar (Peris et al. 2019), martens (*Martes* spp.; Randler et al. 2020), and leopards ( *Panthera pardus* ; du Preez et al. 2014) compared to unbaited sites.

We observed increased nocturnality of black bears at baited sites. Nocturnal use of anthropogenic food sources is common in black bears (Ayres et al. 1986; Zeller et al. 2019), and nocturnality increases due to human disturbance among several carnivore species (Frey et al. 2020; Kautz et al. 2021). However, our results contrast with Bowman et al. (2015) in the same region as our study who observed more diurnal than nocturnal detections of black bears at bait sites. Study methodology may explain this difference; Bowman et al. (2015) described black bear activity patterns using remote cameras whereas our study and that of Zeller et al. (2019) used GPS‐monitored black bears. Remote cameras only recorded activity directly at bait sites while our approach considered all locations within 100 m of bait as active use. It is likely that black bears were not at bait sites during all locations we considered “used,” but instead remained near bait sites to exclude others and optimize foraging. However, a limitation of our study is that we could only assess GPS‐monitored black bears, whereas Bowman et al. (2015) could observe all black bears. Correspondingly, monopolization of bait resources by unmonitored black bears is a factor we could not assess.

Contrary to our predictions, movement metrics did not differ between black bears with or without bait sites in their home ranges. While anthropogenic food subsidies have influenced these metrics among gray wolves ( *Canis lupus* ; Petroelje et al. 2019) and white‐tailed deer (Kilpatrick and Stober 2002), results from studies among black bears are equivocal. Female black bear movements in Alberta, Canada were minimally influenced by baiting and male harvest (Czetwertynski 2008). Diversionary feeding near Lake Tahoe, USA resulted in black bears shifting their activity away from human refuse, but feeding was only effective when near (< 1 km) unintentional anthropogenic food subsidies (Stringham and Bryant 2015). Results among brown bears are similarly variable; individuals in Yellowstone National Park, USA, for example, shifted their ranges to use gut piles from hunter‐harvested elk ( *Cervus canadensis* ) (Ruth et al. 2003).

An alternate explanation for similar movement metrics observed between our impact and control groups is the potential ubiquity of anthropogenic food subsidies available to black bears. Our impacted group had access to corn bait targeting white‐tailed deer. Though our bait should have been the only bait available targeting deer, as the legal recreational deer baiting period occurred after our treatment period, baiting black bears for hunting activities was legal during our study. Black bear baiting is much less common than deer baiting, but the legal volume of bait for black bears at each site is much greater (MDNR 2025). These recreational bait sites, in combination with other unmonitored anthropogenic food sources (e.g., recreational feeding, human refuse, livestock carcass dumps), could have resulted in black bears outside our baited area having anthropogenic food available. However, we consider this unlikely to have influenced our results because the presence of unrecorded food sources was equally likely within the baited area. Our estimated use of bait sites by black bears could also be more conservative than the use of bait sites in the absence of alternative anthropogenic food.

Our study was limited by sample size and monitoring duration. We monitored relatively few black bears without access to bait sites, and associated statistical error was therefore high. Though generally balanced between sexes, our small sample size also precluded analyses comparing males and females. Males have generally larger home ranges (Koehler and Pierce 2003) and may be more prone to visit bait sites (Garshelis and Noyce 2006). Because our monitoring period was limited to July and August, our results describe only the summer period when black bears are more prone to using human resources (McFadden‐Hiller et al. 2016). As such, the application of bait during other seasonal periods may produce differing results.

Appropriate regulation of bait use by recreational stakeholders warrants thoughtful consideration. Though difficult to quantify (Dunkley and Cattet 2003), wildlife baiting is of great economic importance. Wildlife watchers and hunters spent > $6.3 billion on direct food subsidies and bait in 2016 (USDOI et al. 2018). Baiting for black bears is prevalent, with more than half of black bears harvested in Wisconsin, USA during 1995–2003 harvested using bait (Treves et al. 2010). Countering potential economic benefits, baiting can promote disease spread leading to population declines and increased expenditures (Ramsey et al. 2014; Sorensen et al. 2014). Further, human–wildlife conflicts cost $22.3 billion annually in the United States and may increase when wildlife become habituated to anthropogenic food sources (Conover 2002; Dubois and Fraser 2013).

Our results indicate black bears substantially increased their use of sites provisioned with bait for white‐tailed deer research but did not exhibit corresponding changes to their broad‐scale movement patterns. Baiting is a common practice used in white‐tailed deer and black bear harvest management (Frawley 2017; Lafferty et al. 2024). Corresponding to our results, we recommend careful consideration of potential detrimental effects to black bears (Dobey et al. 2005), other nontarget species (Selva et al. 2014), and local habitats (Manning and Baltzer 2011) when assessing white‐tailed deer bait applications for research or hunting. We also recommend future work consider nonconsumptive attractants (i.e., scent lures) as less influential alternatives for short‐term attraction of black bears (Iannarilli et al. 2021).

## Author Contributions

**Nathaniel H. Wehr:** conceptualization (equal), data curation (equal), formal analysis (lead), visualization (lead), writing – original draft (lead), writing – review and editing (lead). **Nicholas L. Fowler:** conceptualization (equal), data curation (equal), writing – review and editing (equal). **Todd M. Kautz:** conceptualization (equal), data curation (equal), writing – review and editing (equal). **Tyler R. Petroelje:** conceptualization (equal), data curation (equal), writing – review and editing (equal). **Dean E. Beyer Jr:** conceptualization (equal), data curation (equal), writing – review and editing (equal). **Jerrold L. Belant:** conceptualization (lead), data curation (lead), funding acquisition (lead), project administration (lead), writing – review and editing (equal).

## Funding

This work was supported by the Michigan Department of Natural Resources, Safari Club International Foundation, Federal Aid in Wildlife Restoration Act, Boone and Crockett Program in Wildlife Conservation at Michigan State University, and Safari Club International Michigan Involvement Committee.

## Conflicts of Interest

The authors declare no conflicts of interest.

## Supporting information


**Appendix S1:** ece373015‐sup‐0001‐AppendixS1.pdf.

## Data Availability

Data used for this study were made available to reviewers during the peer review process but have not been made publicly available in accordance with Michigan Department of Natural Resources (MDNR) policy. Data may be acquired via reasonable request to the MDNR. R code used for this study is available in Appendix S1.
