# Supplementary material for: White‐Tailed Deer Baiting Altered Black Bear Site Use but Not Movements or Range Size
Source: Ecol Evol. 2026 Feb 12;16(2):e73015. doi: 10.1002/ece3.73015 (PMC12895461; doi:10.1002/ece3.73015)
Supplement: Supplementary file 1 — Appendix S1: ece373015‐sup‐0001‐AppendixS1.pdf. [file ECE3-16-e73015-s001.pdf]

Appendix 1:
White-tailed deer baiting altered black bear site use but not movements or range size

Nathaniel H. Wehr   Nicholas L. Fowler   Todd M. Kautz   Tyler R. Petroelje
Dean E. Beyer Jr.   Jerrold L. Belant

Introduction

This code was used to assess black bears' use of corn bait sites intended to attract white-tailed deer. To conduct this analysis, we used spatial data derived from GPS monitoring of black bears in the Upper Peninsula of Michigan, USA during 2013–2015 and 2017–2019.

Part 1: Bait Site Recursion

We used the Recurse package to calculate metrics of bears visiting bait sites and control sites during the pretreatment and treatment periods.

```
# Load packages
library(dplyr)
library(sf)
library(lubridate)
library(recurse)

# Set working directory
setwd("~/All Files/R Working Directory/Bear Bait Use")

# Load dataframes
load("Bear Data Edits - V5 NHW.rda")
Bait <- read.csv("Bait_Sites.csv")
Control <- read.csv("Unbaited_Sites.csv")

# Format data for recurse package
RData <- st_as_sf(BD3, coords = c("location.long", "location.lat"),
                  crs = "+proj=longlat +datum=NAD83")
RData <- st_transform(RData, crs = 26916)
RData <- cbind(RData, st_coordinates(RData))
RData$geometry <- NULL
RData$timestamp <- ymd_hms(RData$timestamp)
RData$Year <- substr(RData$BearYear, 7, 10)

# Subset bear movements to specific time periods and variables
B2013 <- subset(RData, Year == 2013)
B2014 <- subset(RData, Year == 2014)
```

```r
B2015 <- subset(RData, Year == 2015)
B2017 <- subset(RData, Year == 2017)
B2018 <- subset(RData, Year == 2018)
B2019 <- subset(RData, Year == 2019)
BB2013 <- subset(B2013, Period == "Before")
BB2014 <- subset(B2014, Period == "Before")
BB2015 <- subset(B2015, Period == "Before")
BB2017 <- subset(B2017, Period == "Before")
BB2018 <- subset(B2018, Period == "Before")
BB2019 <- subset(B2019, Period == "Before")
BA2013 <- subset(B2013, Period == "After")
BA2014 <- subset(B2014, Period == "After")
BA2015 <- subset(B2015, Period == "After")
BA2017 <- subset(B2017, Period == "After")
BA2018 <- subset(B2018, Period == "After")
BA2019 <- subset(B2019, Period == "After")
BB2013 <- dplyr::select(BB2013, c(X, Y, timestamp, BearYear))
BB2014 <- dplyr::select(BB2014, c(X, Y, timestamp, BearYear))
BB2015 <- dplyr::select(BB2015, c(X, Y, timestamp, BearYear))
BB2017 <- dplyr::select(BB2017, c(X, Y, timestamp, BearYear))
BB2018 <- dplyr::select(BB2018, c(X, Y, timestamp, BearYear))
BB2019 <- dplyr::select(BB2019, c(X, Y, timestamp, BearYear))
BA2013 <- dplyr::select(BA2013, c(X, Y, timestamp, BearYear))
BA2014 <- dplyr::select(BA2014, c(X, Y, timestamp, BearYear))
BA2015 <- dplyr::select(BA2015, c(X, Y, timestamp, BearYear))
BA2017 <- dplyr::select(BA2017, c(X, Y, timestamp, BearYear))
BA2018 <- dplyr::select(BA2018, c(X, Y, timestamp, BearYear))
BA2019 <- dplyr::select(BA2019, c(X, Y, timestamp, BearYear))
BB2013 <- rename(BB2013, x = X, y = Y, ID = BearYear)
BB2014 <- rename(BB2014, x = X, y = Y, ID = BearYear)
BB2015 <- rename(BB2015, x = X, y = Y, ID = BearYear)
BB2017 <- rename(BB2017, x = X, y = Y, ID = BearYear)
BB2018 <- rename(BB2018, x = X, y = Y, ID = BearYear)
BB2019 <- rename(BB2019, x = X, y = Y, ID = BearYear)
BA2013 <- rename(BA2013, x = X, y = Y, ID = BearYear)
BA2014 <- rename(BA2014, x = X, y = Y, ID = BearYear)
BA2015 <- rename(BA2015, x = X, y = Y, ID = BearYear)
BA2017 <- rename(BA2017, x = X, y = Y, ID = BearYear)
BA2018 <- rename(BA2018, x = X, y = Y, ID = BearYear)
BA2019 <- rename(BA2019, x = X, y = Y, ID = BearYear)
rm(B2013, B2014, B2015, B2017, B2018, B2019, BD3)

# Subset bait sites to specific years
I2013 <- subset(Bait, Year == 2013)
I2014 <- subset(Bait, Year == 2014)
I2015 <- subset(Bait, Year == 2015)
I2017 <- subset(Bait, Year == 2017)
I2018 <- subset(Bait, Year == 2018)
I2019 <- subset(Bait, Year == 2019)
I2013 <- dplyr::select(I2013, c(Easting, Northing))
I2014 <- dplyr::select(I2014, c(Easting, Northing))
I2015 <- dplyr::select(I2015, c(Easting, Northing))
I2017 <- dplyr::select(I2017, c(Easting, Northing))
```

```
I2018 <- dplyr::select(I2018, c(Easting, Northing))
I2019 <- dplyr::select(I2019, c(Easting, Northing))

# Subset control sites to specific years
Control$Area <- substr(Control$ID, 1, 2)
C2013 <- subset(Control, Area == "CF")
C2014 <- subset(Control, Area == "CF")
C2015 <- subset(Control, Area == "CF")
C2017 <- subset(Control, Area == "NB")
C2018 <- subset(Control, Area == "NB")
C2019 <- subset(Control, Area == "NB")
C2013 <- dplyr::select(C2013, c(Easting, Northing))
C2014 <- dplyr::select(C2014, c(Easting, Northing))
C2015 <- dplyr::select(C2015, c(Easting, Northing))
C2017 <- dplyr::select(C2017, c(Easting, Northing))
C2018 <- dplyr::select(C2018, c(Easting, Northing))
C2019 <- dplyr::select(C2019, c(Easting, Northing))

# Record visits to bait sites
#create an empty dataframe
All_Results <- data.frame(matrix(ncol = 3, nrow = 1))
#name columns
colnames(All_Results) <- c("Treatment", "Revisits", "Residence_Time")
All_Stats <- data.frame(matrix(ncol = 10, nrow = 1)) # create an empty dataframe
colnames(All_Stats) <- c("id", "x", "y", "coordIdx",
                         "visitIdx", "entranceTime", "exitTime",
                         "timeInside", "timeSinceLastVisit", "Treatment")
##before control
for(i in c(2013, 2014, 2015, 2017, 2018, 2019)){
  # recurse package assessment
  locVisit <- getRecursionsAtLocations(get(paste0("BB", i)),
                                       get(paste0("C", i)), 100)
   # create an empty dataframe
  Results <- data.frame(matrix(ncol = 3, nrow = length(locVisit$revisits)))
   # name columns
  colnames(Results) <- c("Treatment", "Revisits", "Residence_Time")
  Results$Treatment <- paste0("BC_", i) # save treatment
  Results$Revisits <- locVisit$revisits # save revisits count
  Results$Residence_Time <- locVisit$residenceTime # save residence time
  Stats <- locVisit$revisitStats # extract recursion metrics from outputs
  if(nrow(Stats) > 0){Stats$Treatment <- paste0("BC_", i)} # save treatment
  All_Results <- rbind(All_Results, Results) # save results
   # save recursion stats
  if(nrow(Stats) > 0 ){All_Stats <- rbind(All_Stats, Stats)}
}
##before impact
for(i in c(2013, 2014, 2015, 2017, 2018, 2019)){
   # recurse package assessment
  locVisit <- getRecursionsAtLocations(get(paste0("BB", i)),
                                       get(paste0("I", i)), 100)
   # create an empty dataframe
  Results <- data.frame(matrix(ncol = 3, nrow = length(locVisit$revisits)))
   # name columns
```

```
  colnames(Results) <- c("Treatment", "Revisits", "Residence_Time")
  Results$Treatment <- paste0("BI_", i) # save treatment
  Results$Revisits <- locVisit$revisits # save revisits count
  Results$Residence_Time <- locVisit$residenceTime # save residence time
  Stats <- locVisit$revisitStats # extract recursion metrics from outputs
  if(nrow(Stats) > 0){Stats$Treatment <- paste0("BI_", i)} # save treatment
  All_Results <- rbind(All_Results, Results) # save results
   # save recursion stats
  if(nrow(Stats) > 0 ){All_Stats <- rbind(All_Stats, Stats)}
}
##after control
for(i in c(2013, 2014, 2015, 2017, 2018, 2019)){
   # recurse package assessment
  locVisit <- getRecursionsAtLocations(get(paste0("BA", i)),
                                       get(paste0("C", i)), 100)
   # create an empty dataframe
  Results <- data.frame(matrix(ncol = 3, nrow = length(locVisit$revisits)))
   # name columns
  colnames(Results) <- c("Treatment", "Revisits", "Residence_Time")
  Results$Treatment <- paste0("AC_", i) # save treatment
  Results$Revisits <- locVisit$revisits # save revisits count
  Results$Residence_Time <- locVisit$residenceTime # save residence time
  Stats <- locVisit$revisitStats # extract recursion metrics from outputs
  if(nrow(Stats) > 0){Stats$Treatment <- paste0("AC_", i)} # save treatment
  All_Results <- rbind(All_Results, Results) # save results
   # save recursion stats
  if(nrow(Stats) > 0 ){All_Stats <- rbind(All_Stats, Stats)}
}
##after impact
for(i in c(2013, 2014, 2015, 2017, 2018, 2019)){
   # recurse package assessment
  locVisit <- getRecursionsAtLocations(get(paste0("BA", i)),
                                       get(paste0("I", i)), 100)
   # create an empty dataframe
  Results <- data.frame(matrix(ncol = 3, nrow = length(locVisit$revisits)))
   # name columns
  colnames(Results) <- c("Treatment", "Revisits", "Residence_Time")
  Results$Treatment <- paste0("AI_", i) # save treatment
  Results$Revisits <- locVisit$revisits # save revisits count
  Results$Residence_Time <- locVisit$residenceTime # save residence time
  Stats <- locVisit$revisitStats # extract recursion metrics from outputs
  if(nrow(Stats) > 0){Stats$Treatment <- paste0("AI_", i)} # save treatment
  All_Results <- rbind(All_Results, Results) # save results
   # save recursion stats
  if(nrow(Stats) > 0 ){All_Stats <- rbind(All_Stats, Stats)}
}

# Save progress
save(All_Results, All_Stats, RData, file = "Recursion Results - V2.rda")
rm(BA2013, BA2014, BA2015, BA2017, BA2018, BA2019, Bait,
   BB2013, BB2014, BB2015, BB2017, BB2018, BB2019,
   C2013, C2014, C2015, C2017, C2018, C2019, Control, i,
   I2013, I2014, I2015, I2017, I2018, I2019, locVisit, Results, Stats)
```

```
rm(All_Results, All_Stats, RData)
```

Part 2: Movement Metrics

We calculated movement metrics for each bear (home range area, core range area, step lengths, turning angles) during the pretreatment and treatment periods within and outside the baited study area.

```
# Load Libraries
library(dplyr)
library(adehabitatHR)
library(sf)

# Set working directory
setwd("~/All Files/R Working Directory/Bear Bait Use")

# Load dataframes
load("Recursion Results - V2.rda")

# Generate subsets
BD3 <- RData
External <- subset(BD3, Access == 0)
BC <- subset(External, Period == "Before")
AC <- subset(External, Period == "After")
Access <- subset(BD3, Access == 1)
BI <- subset(Access, Period == "Before")
AI <- subset(Access, Period == "After")
rm(Access, External, BD3, RData)

# Calculate movement metrics for four subgroups
##before control
 # create an empty dataframe
Results <- data.frame(matrix(ncol = 5, nrow = length(unique(BC$Name))))
colnames(Results) <- c("ID", "HR", "CR", "SL", "TA") # name columns
for(i in 1:length(unique(BC$Name))){ # cycle through each bear
  Bear <- subset(BC, BC$Name == unique(BC$Name)[i]) # subset to each bear
  Results$ID[i] <- Bear$Name[1] # add bear ID to results
  Bear2 <- dplyr::select(Bear, X, Y) # select just bear location data
  Bear2 <- SpatialPoints(Bear2) # convert to spatial points object
  HR <- kernelUD(Bear2) # use kernel density estimation to estimate a home range
  HR_S <- getverticeshr(HR, percent = 95) # generates outline for 95% home range
  HR_S <- st_as_sf(HR_S) # Convert to spatial feature
  st_crs(HR_S) <- 26916 # set CRS
  st_write(HR_S, paste0("Results/KDE95_", unique(BC$Name)[i], ".shp"),
           delete_layer = TRUE, quiet = TRUE) # Generate shapefile
  # record home range size
  Results$HR[i] <- kernel.area(HR, percent = 95, unout = "km2")
   # record core range size
  Results$CR[i] <- kernel.area(HR, percent = 50, unout = "km2")
   # convert bear to trajectory object
  Bear3 <- as.ltraj(xy = Bear[,c("X","Y")],
                    date = Bear$timestamp, id = Bear$Name)
  Results$SL[i] <- median(na.omit(Bear3[[1]]$dist)) # record median step length
```

```
  # record median turning angle (ie tortuosity)
  Results$TA[i] <- median(abs(na.omit(Bear3[[1]]$rel.angle)))
}
Results$Treatment <- "BC"
BC <- Results
##after control
Results <- data.frame(matrix(ncol = 5, nrow = length(unique(AC$Name))))
colnames(Results) <- c("ID", "HR", "CR", "SL", "TA")
for(i in 1:length(unique(AC$Name))){
  Bear <- subset(AC, AC$Name == unique(AC$Name)[i])
  Results$ID[i] <- Bear$Name[1]
  Bear2 <- dplyr::select(Bear, X, Y)
  Bear2 <- SpatialPoints(Bear2)
  HR <- kernelUD(Bear2)
  HR <- kernelUD(Bear2) # use kernel density estimation to estimate a home range
  HR_S <- getverticeshr(HR, percent = 95) # generates outline for 95% home range
  HR_S <- st_as_sf(HR_S) # Convert to spatial feature
  st_crs(HR_S) <- 26916 # set CRS
  st_write(HR_S, paste0("Results/KDE95_", unique(AC$Name)[i], ".shp"),
           delete_layer = TRUE, quiet = TRUE) # Generate shapefile
  Results$HR[i] <- kernel.area(HR, percent = 95, unout = "km2")
  Results$CR[i] <- kernel.area(HR, percent = 50, unout = "km2")
  Bear3 <- as.ltraj(xy = Bear[,c("X","Y")],
                    date = Bear$timestamp, id = Bear$Name)
  Results$SL[i] <- median(na.omit(Bear3[[1]]$dist))
  Results$TA[i] <- median(abs(na.omit(Bear3[[1]]$rel.angle)))
}
Results$Treatment <- "AC"
AC <- Results
##before impact
Results <- data.frame(matrix(ncol = 5, nrow = length(unique(BI$Name))))
colnames(Results) <- c("ID", "HR", "CR", "SL", "TA")
for(i in 1:length(unique(BI$Name))){
  Bear <- subset(BI, BI$Name == unique(BI$Name)[i])
  Results$ID[i] <- Bear$Name[1]
  Bear2 <- dplyr::select(Bear, X, Y)
  Bear2 <- SpatialPoints(Bear2)
  HR <- kernelUD(Bear2)
  # use kernel density estimation to estimate a home range distribution
  HR <- kernelUD(Bear2)
  HR_S <- getverticeshr(HR, percent = 95) # generates outline for 95% home range
  HR_S <- st_as_sf(HR_S) # Convert to spatial feature
  st_crs(HR_S) <- 26916 # set CRS
  st_write(HR_S, paste0("Results/KDE95_", unique(BI$Name)[i], ".shp"),
           delete_layer = TRUE, quiet = TRUE) # Generate shapefile
  Results$HR[i] <- kernel.area(HR, percent = 95, unout = "km2")
  Results$CR[i] <- kernel.area(HR, percent = 50, unout = "km2")
  Bear3 <- as.ltraj(xy = Bear[,c("X","Y")],
                    date = Bear$timestamp, id = Bear$Name)
  Results$SL[i] <- median(na.omit(Bear3[[1]]$dist))
  Results$TA[i] <- median(abs(na.omit(Bear3[[1]]$rel.angle)))
}
Results$Treatment <- "BI"
```

```
BI <- Results
##after impact
Results <- data.frame(matrix(ncol = 5, nrow = length(unique(AI$Name))))
colnames(Results) <- c("ID", "HR", "CR", "SL", "TA")
for(i in 1:length(unique(AI$Name))){
  Bear <- subset(AI, AI$Name == unique(AI$Name)[i])
  Results$ID[i] <- Bear$Name[1]
  Bear2 <- dplyr::select(Bear, X, Y)
  Bear2 <- SpatialPoints(Bear2)
  HR <- kernelUD(Bear2)
  HR <- kernelUD(Bear2)
  HR_S <- getverticeshr(HR, percent = 95) # generates outline for 95% home range
  HR_S <- st_as_sf(HR_S) # Convert to spatial feature
  st_crs(HR_S) <- 26916 # set CRS
  st_write(HR_S, paste0("Results/KDE95_", unique(AI$Name)[i], ".shp"),
           delete_layer = TRUE, quiet = TRUE) # Generate shapefile
  Results$HR[i] <- kernel.area(HR, percent = 95, unout = "km2")
  Results$CR[i] <- kernel.area(HR, percent = 50, unout = "km2")
  Bear3 <- as.ltraj(xy = Bear[,c("X","Y")],
                    date = Bear$timestamp, id = Bear$Name)
  Results$SL[i] <- median(na.omit(Bear3[[1]]$dist))
  Results$TA[i] <- median(abs(na.omit(Bear3[[1]]$rel.angle)))
}
Results$Treatment <- "AI"
AI <- Results

# Combine results
Movement_Results <- rbind(BC, AC, BI, AI)

# Remove extra objects
rm(AC, AI, BC, BI, Bear, Bear2, Bear3, HR, Results, i)

# Apply IDs
MR <- Movement_Results
MR$BearYear <- substr(MR$ID, 1, 10)

# Save progress
Movement_Results <- MR
save(Movement_Results, All_Results, All_Stats, file = "All Results - V1.rda")
rm(Movement_Results, All_Results, All_Stats, MR, HR_S)
```

Part 3: BACI Analyses

We used a standard Before-After-Control-Impact (BACI) approach (Smith EP. 2002. BACI design. Encyclopedia of Environmetrics). We conducted these tests on the various movement and recursion metrics assessed for bears and bait sites respectively. We used the gamma log-link function for home range and core range to account for individuals with abnormally large ranges, and we used the negative binomial log-link functions for revisits and residence time (Harig F. 2024. DHARMa. Vignette).

```
# Load libraries
library(glmmTMB)
library(dplyr)
```

```r
library(lubridate)
library(DHARMa)
library(circular)
library(overlap)

# Load data
setwd("~/All Files/R Working Directory/Bear Bait Use")
load("All Results - V1.rda")

# Modify Movement Results
MR <- Movement_Results
MR$Period <- substr(MR$Treatment, 1, 1)
MR$Site <- substr(MR$Treatment, 2, 2)

# Home Range Size
Model1 <- glmmTMB(HR ~ Period + Site + Period:Site + (1|BearYear),
                  data = MR, family = Gamma(link = "log"))
summary(Model1)
```

```
##  Family: Gamma  ( log )
## Formula:          HR ~ Period + Site + Period:Site + (1 | BearYear)
## Data: MR
##
##      AIC      BIC   logLik deviance df.resid
##    974.5    988.8   -481.2    962.5       74
##
## Random effects:
##
## Conditional model:
##  Groups   Name        Variance Std.Dev.
##  BearYear (Intercept) 1.25     1.118
## Number of obs: 80, groups:  BearYear, 40
##
## Dispersion estimate for Gamma family (sigma^2): 0.928
##
## Conditional model:
##                Estimate Std. Error z value Pr(>|z|)
## (Intercept)      4.4428     0.5720   7.766 8.07e-15 ***
## PeriodB         -0.4337     0.5802  -0.747    0.455
## SiteI            0.7331     0.6312   1.161    0.245
## PeriodB:SiteI   -0.2601     0.6427  -0.405    0.686
## ---
## Signif. codes:  0 '***' 0.001 '**' 0.01 '*' 0.05 '.' 0.1 ' ' 1
```

```r
testDispersion(Model1)
```

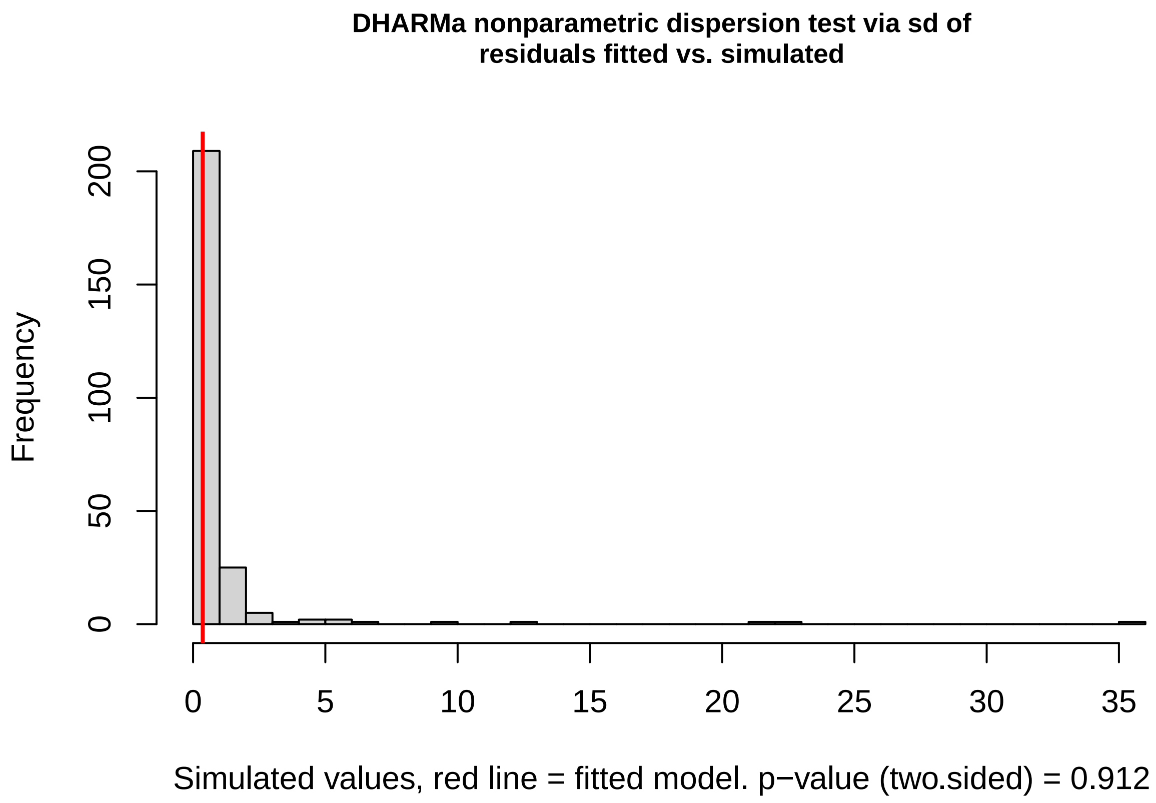


```
##
##  DHARMa nonparametric dispersion test via sd of residuals fitted vs.
##  simulated
##
## data:  simulationOutput
## dispersion = 0.36782, p-value = 0.912
## alternative hypothesis: two.sided
```

```
# Core Range Size
Model2 <- glmmTMB(CR ~ Period + Site + Period:Site + (1|BearYear),
                  data = MR, family = Gamma(link = "log"))
summary(Model2)
```

```
##  Family: Gamma  ( log )
## Formula:          CR ~ Period + Site + Period:Site + (1 | BearYear)
## Data: MR
##
##      AIC      BIC   logLik deviance df.resid
##    708.8    723.1   -348.4    696.8       74
##
## Random effects:
##
## Conditional model:
##  Groups   Name        Variance Std.Dev.
##  BearYear (Intercept) 1.323    1.15
```

```
## Number of obs: 80, groups:  BearYear, 40
##
## Dispersion estimate for Gamma family (sigma^2): 0.832
##
## Conditional model:
##                Estimate Std. Error z value Pr(>|z|)
## (Intercept)      2.6335     0.5552   4.743  2.1e-06 ***
## PeriodB         -0.1624     0.5316  -0.305    0.760
## SiteI            0.7514     0.6161   1.220    0.223
## PeriodB:SiteI   -0.3270     0.5930  -0.551    0.581
## ---
## Signif. codes:  0 '***' 0.001 '**' 0.01 '*' 0.05 '.' 0.1 ' ' 1
```

```r
testDispersion(Model2)
```

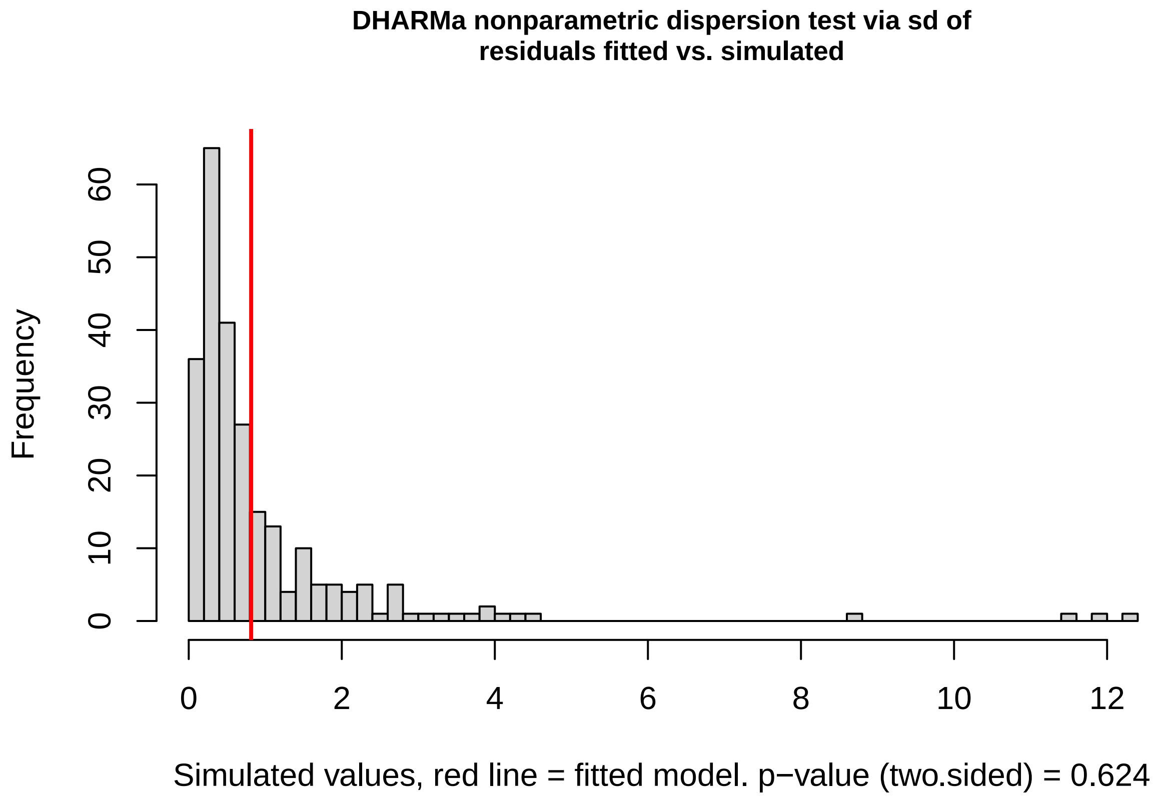


```
##
##  DHARMa nonparametric dispersion test via sd of residuals fitted vs.
##  simulated
##
## data:  simulationOutput
## dispersion = 0.83163, p-value = 0.624
## alternative hypothesis: two.sided
```

```r
# Step Lengths
Model3 <- glmmTMB(SL ~ Period + Site + Period:Site + (1|BearYear),
                  data = MR, family = gaussian())
summary(Model3)
```

```
##  Family: gaussian  ( identity )
## Formula:          SL ~ Period + Site + Period:Site + (1 | BearYear)
## Data: MR
##
##      AIC      BIC   logLik deviance df.resid
##    662.4    676.7   -325.2    650.4       74
##
## Random effects:
##
## Conditional model:
##  Groups   Name        Variance Std.Dev.
##  BearYear (Intercept) 104.6    10.23
##  Residual             120.1    10.96
## Number of obs: 80, groups:  BearYear, 40
##
## Dispersion estimate for gaussian family (sigma^2):  120
##
## Conditional model:
##                Estimate Std. Error z value Pr(>|z|)
## (Intercept)      31.702      5.300   5.982 2.21e-09 ***
## PeriodB           6.859      5.480   1.252   0.2107
## SiteI             1.756      5.925   0.296   0.7670
## PeriodB:SiteI    10.701      6.127   1.746   0.0807 .
## ---
## Signif. codes:  0 '***' 0.001 '**' 0.01 '*' 0.05 '.' 0.1 ' ' 1
```

```r
testDispersion(Model3)
```

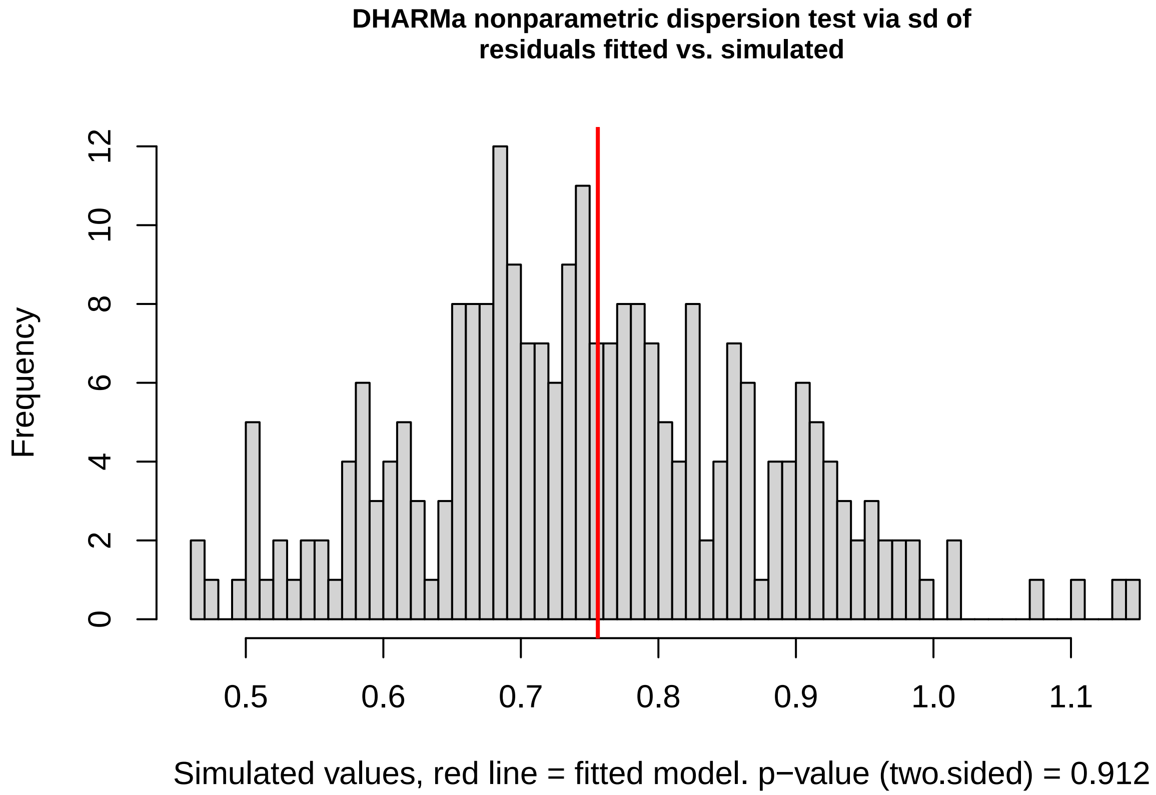


```
##
##  DHARMa nonparametric dispersion test via sd of residuals fitted vs.
##  simulated
##
## data:  simulationOutput
## dispersion = 1.0067, p-value = 0.912
## alternative hypothesis: two.sided
```

```
# Turning Angles
Model4 <- glmmTMB(TA ~ Period + Site + Period:Site + (1|BearYear),
                  data = MR, family = gaussian())
summary(Model4)
```

```
##  Family: gaussian  ( identity )
## Formula:          TA ~ Period + Site + Period:Site + (1 | BearYear)
## Data: MR
##
##      AIC      BIC   logLik deviance df.resid
##    -79.9    -65.6     45.9    -91.9       74
##
## Random effects:
##
## Conditional model:
##  Groups   Name        Variance Std.Dev.
##  BearYear (Intercept) 0.01056  0.1028
```

```
##  Residual             0.01080  0.1039
## Number of obs: 80, groups:  BearYear, 40
##
## Dispersion estimate for gaussian family (sigma^2): 0.0108
##
## Conditional model:
##                Estimate Std. Error z value Pr(>|z|)
## (Intercept)     1.40757    0.05168  27.237   <2e-16 ***
## PeriodB       -0.07876    0.05197  -1.516   0.1296
## SiteI         -0.10475    0.05778  -1.813   0.0698 .
## PeriodB:SiteI -0.06152    0.05810  -1.059   0.2897
## ---
## Signif. codes:  0 '***' 0.001 '**' 0.01 '*' 0.05 '.' 0.1 ' ' 1
```

```r
testDispersion(Model4)
```

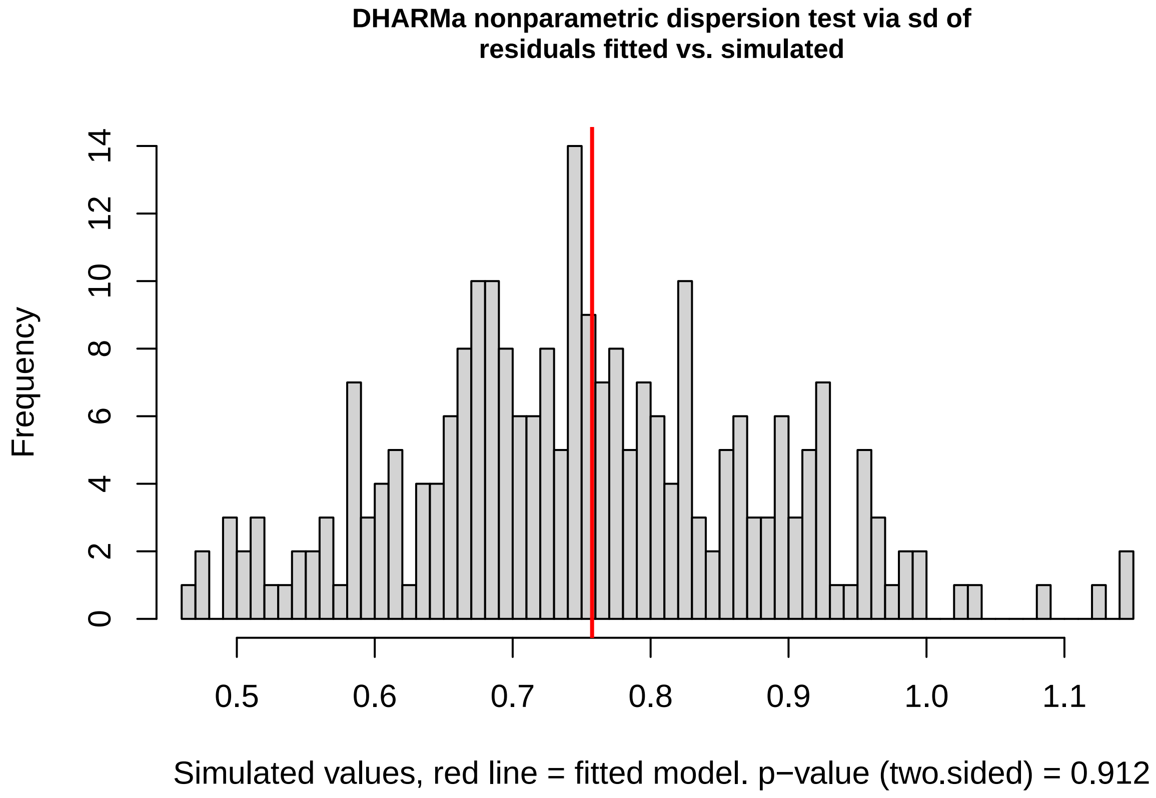


```
##
##  DHARMa nonparametric dispersion test via sd of residuals fitted vs.
##  simulated
##
## data:  simulationOutput
## dispersion = 1.0063, p-value = 0.912
## alternative hypothesis: two.sided
```

```r
# Clean environment
rm(Model1, Model2, Model3, Model4, Movement_Results)

# Modify Bait Site Results
RR <- na.omit(All_Results)
RR$Period <- substr(RR$Treatment, 1, 1)
RR$Site <- substr(RR$Treatment, 2, 2)
RR0 <- subset(RR, Revisits > 0)

# Revisits w/o Zeros
Model5 <- glmmTMB(Revisits ~ Period + Site + Period:Site + (1|Treatment),
                  data = RR0, family = nbinom2(link = "log"))
summary(Model5)
```

```
##  Family: nbinom2  ( log )
## Formula:          Revisits ~ Period + Site + Period:Site + (1 | Treatment)
## Data: RR0
##
##      AIC      BIC   logLik deviance df.resid
##    356.8    371.9   -172.4    344.8       86
##
## Random effects:
##
## Conditional model:
##  Groups    Name        Variance Std.Dev.
##  Treatment (Intercept) 0.07533  0.2745
## Number of obs: 92, groups:  Treatment, 17
##
## Dispersion parameter for nbinom2 family ():  4.1
##
## Conditional model:
##               Estimate Std. Error z value Pr(>|z|)
## (Intercept)     0.1281     0.2716   0.472  0.63708
## PeriodB         0.4079     0.3528   1.156  0.24754
## SiteI           1.3921     0.3458   4.025 5.69e-05 ***
## PeriodB:SiteI  -1.4225     0.4906  -2.899  0.00374 **
## ---
## Signif. codes:  0 '***' 0.001 '**' 0.01 '*' 0.05 '.' 0.1 ' ' 1
```

```r
testDispersion(Model5)
```

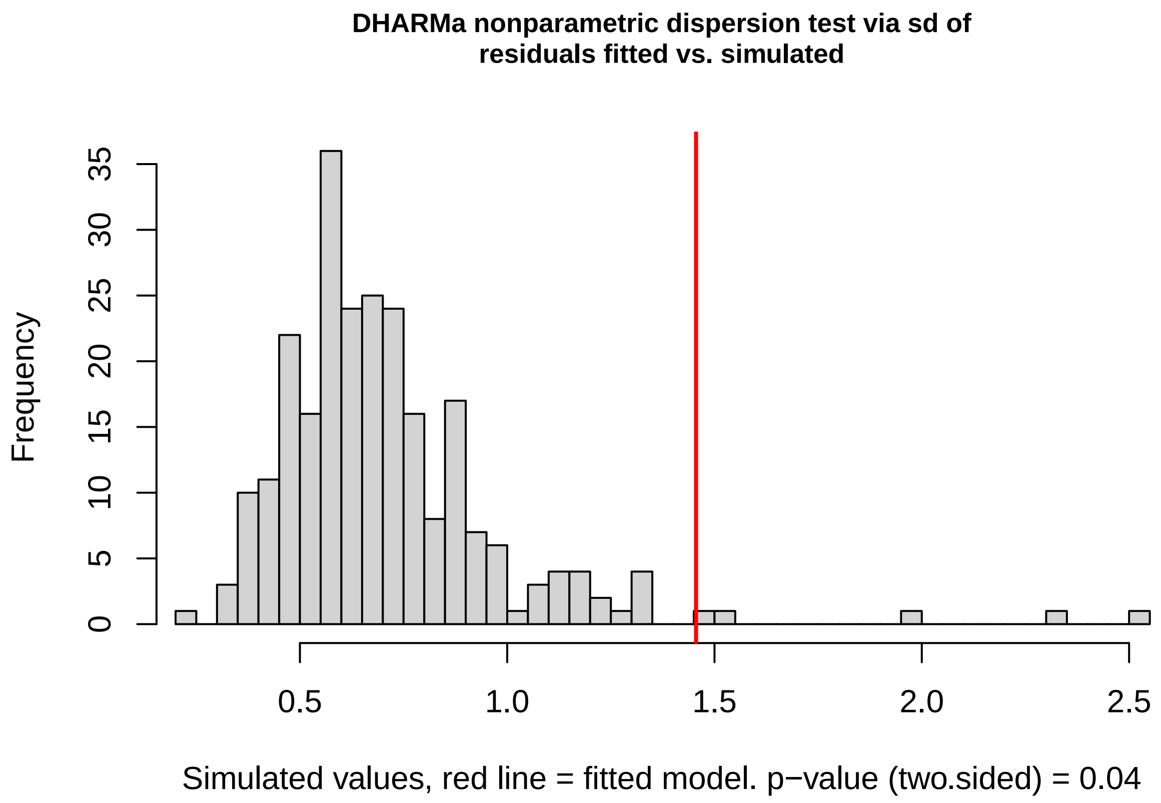


```
##
##  DHARMa nonparametric dispersion test via sd of residuals fitted vs.
##  simulated
##
## data:  simulationOutput
## dispersion = 2.0585, p-value = 0.04
## alternative hypothesis: two.sided
```

```
# Residence Time w/o Zeros
Model6 <- glmmTMB(Residence_Time ~ Period + Site + Period:Site + (1|Treatment),
                  data = RR0, family = nbinom2(link = "log"))
summary(Model6)
```

```
##  Family: nbinom2  ( log )
## Formula:
## Residence_Time ~ Period + Site + Period:Site + (1 | Treatment)
## Data: RR0
##
##      AIC      BIC   logLik deviance df.resid
##    307.1    322.2   -147.5    295.1       86
##
## Random effects:
##
## Conditional model:
##  Groups    Name        Variance Std.Dev.
```

```
##  Treatment (Intercept) 0.229    0.4786
## Number of obs: 92, groups:  Treatment, 17
##
## Dispersion parameter for nbinom2 family (): 0.65
##
## Conditional model:
##               Estimate Std. Error z value Pr(>|z|)
## (Intercept)    -0.5588     0.5128  -1.090  0.27587
## PeriodB         0.1711     0.6487   0.264  0.79199
## SiteI           2.1483     0.6236   3.445  0.00057 ***
## PeriodB:SiteI  -2.5094     0.9267  -2.708  0.00677 **
## ---
## Signif. codes:  0 '***' 0.001 '**' 0.01 '*' 0.05 '.' 0.1 ' ' 1
```

```r
testDispersion(Model6)
```

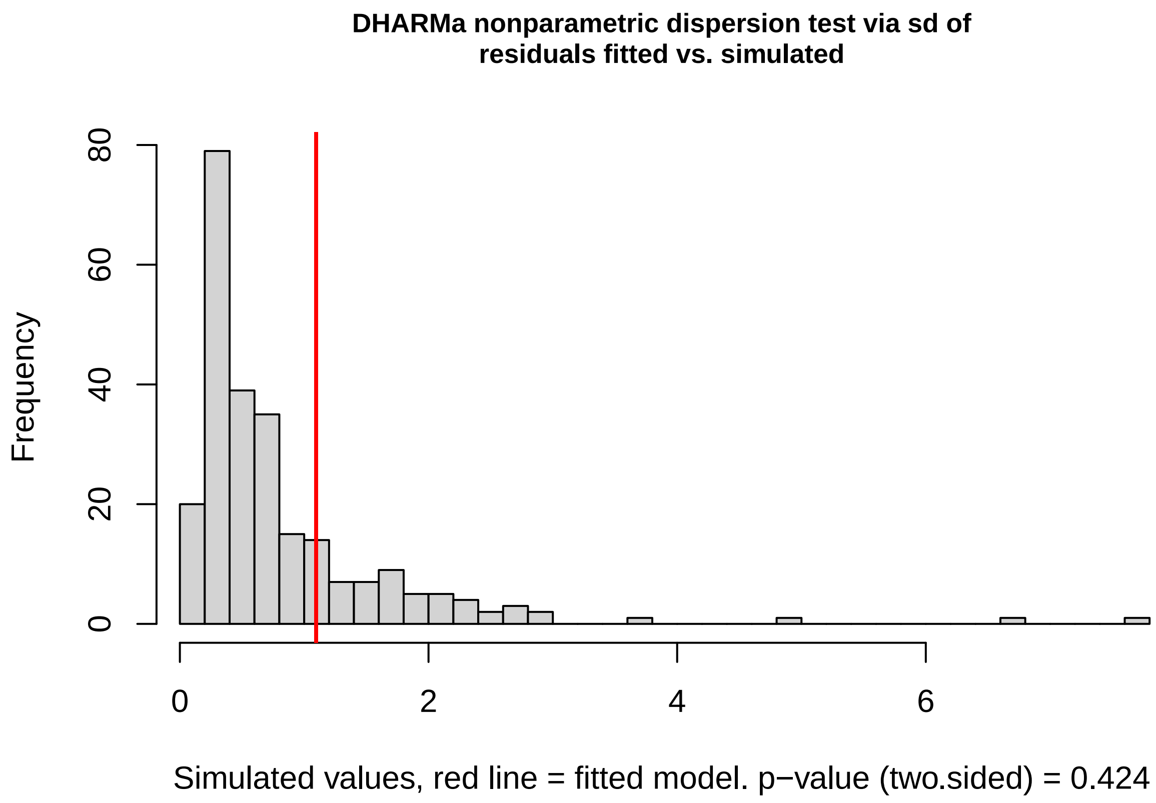


```
##
##  DHARMa nonparametric dispersion test via sd of residuals fitted vs.
##  simulated
##
## data:  simulationOutput
## dispersion = 1.3421, p-value = 0.424
## alternative hypothesis: two.sided
```

```r
# Clean Environment
rm(All_Results, Model5, Model6)

# Modify Bait Site Results
RS <- All_Stats[-1,]
RS$Period <- substr(RS$Treatment, 1, 1)
RS$Site <- substr(RS$Treatment, 2, 2)
RS$ToD <- substr(as.character(as.POSIXct(RS$entranceTime)), 12, 19)
RS <- dplyr::mutate(RS, fractime = hms(ToD) / hms("24:00:00"),
                    RadTime = fractime*2*pi, CosTime = cos(RadTime))
RS$RadTime_centered <- ifelse(RS$RadTime > pi, RS$RadTime - 2*pi, RS$RadTime)
RT <- na.omit(RS)
RT$Days <- RT$timeSinceLastVisit / 24

# Return Rate
Model7 <- glmmTMB(Days ~ Period + Site + Period:Site + (1|id),
                  data = RT, family = Gamma(link = "log"))
summary(Model7)
```

```
##  Family: Gamma  ( log )
## Formula:          Days ~ Period + Site + Period:Site + (1 | id)
## Data: RT
##
##      AIC      BIC   logLik deviance df.resid
##    408.7    426.8   -198.3    396.7      145
##
## Random effects:
##
## Conditional model:
##  Groups Name        Variance Std.Dev.
##  id     (Intercept) 1.35e-09 3.674e-05
## Number of obs: 151, groups:  id, 13
##
## Dispersion estimate for Gamma family (sigma^2): 1.89
##
## Conditional model:
##                Estimate Std. Error z value Pr(>|z|)
## (Intercept)      1.6439     0.9732   1.689   0.0912 .
## PeriodB         -0.1835     1.0289  -0.178   0.8585
## SiteI           -1.4176     0.9815  -1.444   0.1487
## PeriodB:SiteI    1.2716     1.0959   1.160   0.2459
## ---
## Signif. codes:  0 '***' 0.001 '**' 0.01 '*' 0.05 '.' 0.1 ' ' 1
```

```r
testDispersion(Model7)
```

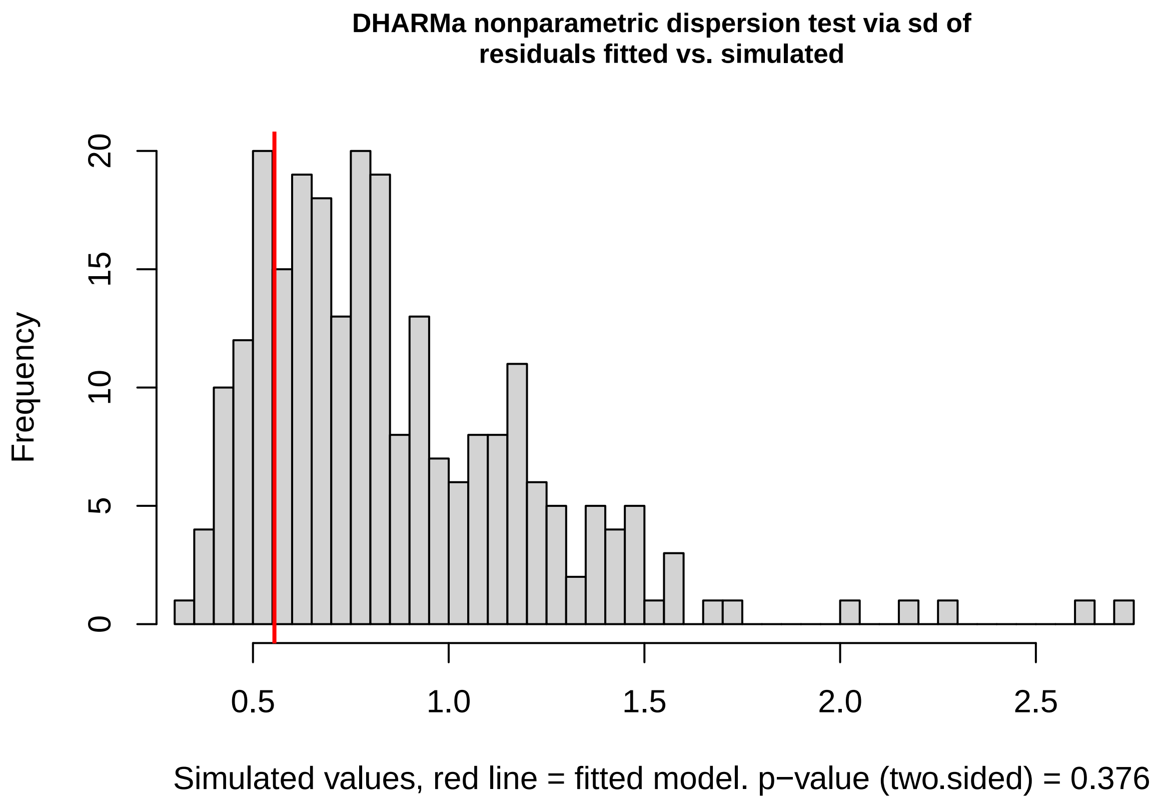


```
##
##  DHARMa nonparametric dispersion test via sd of residuals fitted vs.
##  simulated
##
## data:  simulationOutput
## dispersion = 0.64277, p-value = 0.376
## alternative hypothesis: two.sided
```

```
# Entrance Time
#subset data
RS$Treatment <- paste0(RS$Period, RS$Site)
BC <- subset(RS, Treatment == "BC")
AC <- subset(RS, Treatment == "AC")
BI <- subset(RS, Treatment == "BI")
AI <- subset(RS, Treatment == "AI")

#Watson Test
watson.two.test(BC$RadTime, AC$RadTime)
```

```
##
##      Watson's Two-Sample Test of Homogeneity
##
## Test Statistic: 0.1145
## P-value > 0.10
##
```

```r
watson.two.test(BI$RadTime, AI$RadTime)
```

```
##
##       Watson's Two-Sample Test of Homogeneity
##
## Test Statistic: 0.4392
## P-value < 0.001
##
```

```r
#overlap test Dhat1
overlapEst(BC$RadTime, AC$RadTime, type = "Dhat1")
```

```
##     Dhat1
## 0.6732853
```

```r
overlapEst(BI$RadTime, AI$RadTime, type = "Dhat1")
```

```
##    Dhat1
## 0.648529
```

```r
# Clean environment
rm(All_Stats, Model7)

# Save Progress
save(MR, RR, RR0, RS, RT, file = "Mod Results - V1.rda")
rm(MR, RR, RR0, RS, RT)
```

Part 4: Tables and Figures

In addition to generating the tables and figures for our manuscript, we assessed diel activity in this step because of the necessarr

```r
# Load data
setwd("~/All Files/R Working Directory/Bear Bait Use")
load("Mod Results - V1.rda")

# Libraries
library(ggplot2)
library(gridExtra)
library(overlap)

# GGPlot Theme
mytheme <- theme_bw() +
  theme(panel.grid.major=element_blank(),
        panel.grid.minor=element_blank(),
        axis.text = element_text(size=10),
        axis.title = element_text(size=13),
        plot.title = element_text(hjust = 0.5),
        legend.text = element_text(size=10),
```

```r
        strip.background=element_rect("white"),
        strip.text=element_text(size=12))

# Modify Data
RR0$Treatment <- substr(RR0$Treatment, 1, 2)
RS$Treatment <- substr(RS$Treatment, 1, 2)
RT$Treatment <- substr(RT$Treatment, 1, 2)
RS$TT <- sin(RS$RadTime) + cos(RS$RadTime)

# Table 1
#rather than repeating the below lines 8 times
#I modified and reran them as needed for the different response variables
SubBC <- subset(RS, Treatment == "BC")
median(SubBC$TT)
```

```
## [1] -0.107727
```

```r
sd(SubBC$TT)/sqrt(length(SubBC))
```

```
## [1] 0.1510812
```

```r
SubAC <- subset(RS, Treatment == "AC")
median(SubAC$TT)
```

```
## [1] -0.2982552
```

```r
sd(SubAC$TT)/sqrt(length(SubAC))
```

```
## [1] 0.185502
```

```r
SubBI <- subset(RS, Treatment == "BI")
median(SubBI$TT)
```

```
## [1] -0.5633504
```

```r
sd(SubBI$TT)/sqrt(length(SubBI))
```

```
## [1] 0.1853974
```

```r
SubAI <- subset(RS, Treatment == "AI")
median(SubAI$TT)
```

```
## [1] -0.03393543
```

```r
sd(SubAI$TT)/sqrt(length(SubAI))
```

```
## [1] 0.195884
```

```r
# Figure 2
#correct category order
RRO$Treatment <- factor(RRO$Treatment, levels = c("BC", "AC", "BI", "AI"))
#number of visits
Revisits <- ggplot(data = RRO) +
  aes(y = Revisits, x = Treatment) +
  geom_jitter(aes(color = Treatment), alpha = 0.9) +
  stat_summary(fun = median, geom = "point",
               size = 4, color = "black", shape = 18) +
  mytheme +
  theme(legend.position = "none") +
  ylab("Number of visits") +
  xlab("") +
  scale_x_discrete(labels = c('Control: pretreatment','Control: treatment',
                              'Baited: pretreatment', "Baited: treatment"))
#duration of visits
Duration <- ggplot(data = RRO) +
  aes(y = Residence_Time, x = Treatment) +
  geom_jitter(aes(color = Treatment), alpha = 0.9) +
  stat_summary(fun = median, geom = "point",
               size = 4, color = "black", shape = 18) +
  mytheme +
  theme(legend.position = "none") +
  ylab("Duration of visits (h)") +
  xlab("") +
  scale_x_discrete(labels = c('Control: pretreatment','Control: treatment',
                              'Baited: pretreatment', "Baited: treatment")) +
  ylim(0, 12)
#combine
combo1 <- grid.arrange(Revisits, Duration, nrow = 2)
```

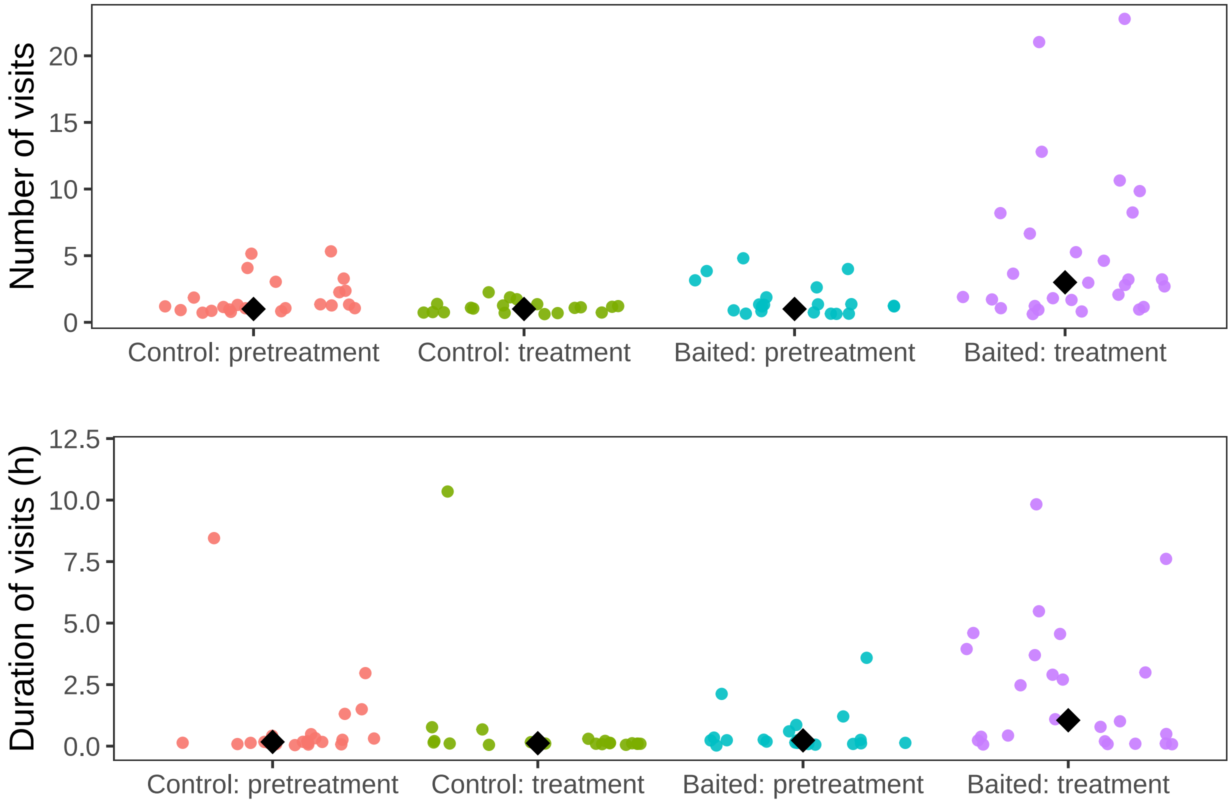


```
#save
ggsave(plot = combo1, width = 6.5, height = 6, dpi = 600,
       filename = "Recursions - V4.jpg")

# Figure 3
#subset data
BC <- subset(RS, Treatment == "BC")
AC <- subset(RS, Treatment == "AC")
BI <- subset(RS, Treatment == "BI")
AI <- subset(RS, Treatment == "AI")

# setup figure
tiff(filename = "Diel - V4.tiff",
     width = 6.5, height = 3, units = 'in', res = 600)
par(mfrow = c(1,2), cex.axis = 0.75, cex.lab = 1.25, mgp = c(1.55, 0.5, 0),
    mai = c(0.25, 0.25, 0.1, 0.1), oma = c(1.25, 1.25, 0, 0))
# control
overlapPlot(BC$RadTime, AC$RadTime, main = '', xlab = '', ylab = '',
            linetype = c(6,1), linecol = c("#F8766D", "#7CAE00"),
            olapcol = "linen", linewidth = c(2,2), ylim = c(0, 0.15))
legend("topleft", c('Control: pretreatment','Control: treatment'),
       lty=c(6,1), lwd = 2, col = c("#F8766D", "#7CAE00"), bty="n", cex = 0.75)
# impact
overlapPlot(BI$RadTime, AI$RadTime, main = '', xlab = '', ylab = '',
            linetype = c(6,1), linecol = c("#00BFC4", "#C77CFF"),
            olapcol = "linen", linewidth = c(2,2), ylim = c(0, 0.15))
```

```r
legend("topleft", c('Baited: pretreatment', "Baited: treatment"),
       lty=c(6,1), lwd = 2, col = c("#00BFC4", "#C77CFF"), bty="n", cex = 0.75)
# axis labels
mtext("Time of day", side = 1, line = 0.25, outer = TRUE)
mtext("Activity density", side = 2, line = 0.25, outer = TRUE)
# turn off figure
dev.off()
```

```
## pdf
##   2
```

```r
# repeat for visual
par(mfrow = c(1,2), cex.axis = 0.75, cex.lab = 1.25, mgp = c(1.55, 0.5, 0),
    mai = c(0.25, 0.25, 0.1, 0.1), oma = c(1.25, 1.25, 0, 0))
# control
overlapPlot(BC$RadTime, AC$RadTime, main = '', xlab = '', ylab = '',
            linetype = c(6,1), linecol = c("#F8766D", "#7CAE00"),
            olapcol = "linen", linewidth = c(2,2), ylim = c(0, 0.15))
legend("topleft", c('Control: pretreatment','Control: treatment'),
       lty=c(6,1), lwd = 2, col = c("#F8766D", "#7CAE00"), bty="n", cex = 0.75)
# impact
overlapPlot(BI$RadTime, AI$RadTime, main = '', xlab = '', ylab = '',
            linetype = c(6,1), linecol = c("#00BFC4", "#C77CFF"),
            olapcol = "linen", linewidth = c(2,2), ylim = c(0, 0.15))
legend("topleft", c('Baited: pretreatment', "Baited: treatment"),
       lty=c(6,1), lwd = 2, col = c("#00BFC4", "#C77CFF"), bty="n", cex = 0.75)
# axis labels
mtext("Time of day", side = 1, line = 0.25, outer = TRUE)
mtext("Activity density", side = 2, line = 0.25, outer = TRUE)
```

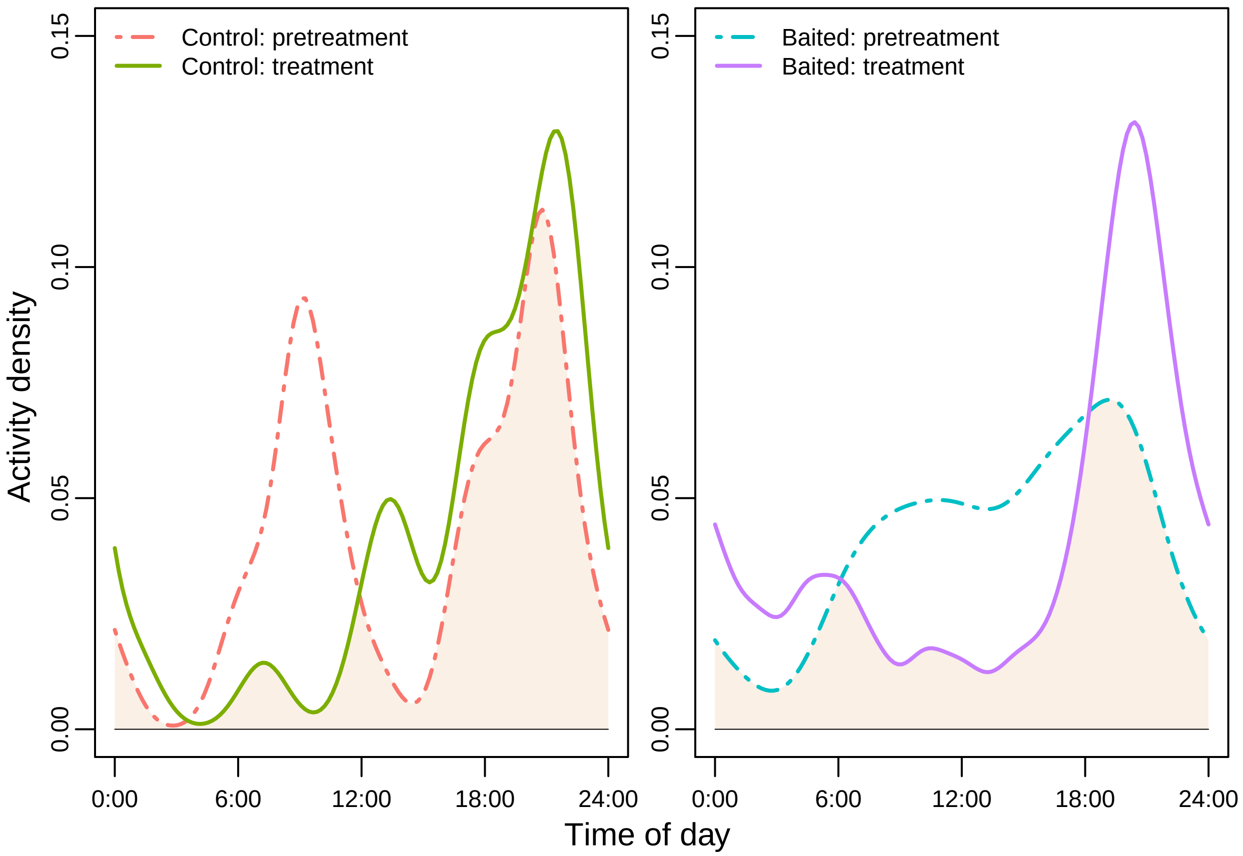
Control: pretreatment
Control: treatment
Baited: pretreatment
Baited: treatment
Activity density
0.00
0.05
0.10
0.15
0:00
6:00
12:00
18:00
24:00
Time of day
